# Fatty acid metabolism after short-term fasting: POMC response and EPA signal maintain homeostasis in tilapia

**DOI:** 10.3389/fendo.2025.1585216

**Published:** 2025-05-09

**Authors:** Xiaozheng Yu, Tiansheng Zhu, Yang Yu, Ran Cai, Meiqing Li, Caiyun Sun, Wensheng Li

**Affiliations:** ^1^ State Key Laboratory of Biocontrol, Institute of Aquatic Economic Animals and Guangdong Province Provincial Engineering Technology Research Center for Healthy Breeding of Important Economic Fish, School of Life Sciences, Sun Yat-Sen University, Guangzhou, China; ^2^ Key Laboratory for Aquatic Economic Animals, Institute of Aquatic Economic Animals, School of Life Sciences, Sun Yat-Sen University, Guangzhou, China

**Keywords:** short-term fasting, POMC, EPA, fatty acid sensing system, tilapia

## Abstract

Detecting and responding to fluctuations in fatty acid levels is crucial for maintaining the homeostasis of fatty acid metabolism. This study examined changes in neuropeptide levels and fatty acid sensing systems in tilapia following 24-hour fasting. Subsequently, an EPA compensation experiment was conducted to examine the regulatory effects of hypothalamic neuropeptides on feeding activity, fatty acid sensing systems activation, and alterations in AMPK and AKT signaling pathways in tilapia. After fasting, the neuropeptide Y signals in the preglomerular nucleus region increased significantly, while the POMC in the lateral tuberal nucleus significantly decreased. There was a significant increase in most long-chain fatty acids, excluding the EPA which declined. Fasting activates fatty acid sensing systems regulated by fatty acid metabolism and mitochondrial activity in the hypothalamus, and those regulated by CD36, mitochondrial activity and PKC in the liver. However, it inhibited systems regulated by fatty acid metabolism and lipoprotein lipase in the liver. Intraperitoneal EPA injection raised *pomc* mRNA levels in the hypothalamus after short-term fasting and curtailed food intake. EPA compensation inhibited the liver fatty acid metabolism, CD36, and mitochondrial activity-related fatty acid sensing systems, and lipoprotein lipase-regulated fatty acid sensing systems in the hypothalamus while activating lipoprotein lipase-regulated fatty acid sensing systems in the liver. Moreover, EPA suppressed the AMPK pathway in both tissues. Following fasting, serum EPA levels decreased, accompanied by lower POMC in the brain and activation of the fatty acid sensing systems in hypothalamus and liver. EPA compensation inhibited the AMPK pathway, increased *pomc* mRNA in the hypothalamus and suppressed food intake as a satiation factor. This research offers insights into how the central nervous system and peripheral tissues respond to fatty acid levels during hunger in tilapia.

## 1 Introduction

Adequate food supply is essential for the survival, activity, growth, and reproduction of animals. However, animals in their natural habitats frequently face the challenge of insufficient food availability. Fish exhibit remarkable adaptability to starvation both in natural environments and controlled experimental conditions (1–4). Most studies on fasting in fish have predominantly focused on assessing the mRNA expression levels of neuropeptides within the brain, rather than investigating their corresponding protein levels in both the brain and blood circulation. Generally, fasting induces an upregulation of feeding-promoting factors while simultaneously downregulating feeding-inhibiting factors across various regions of the fish brain. In orange-spotted grouper (*Epinephelus coioides*) (5), zebrafish (*Danio rerio*) (6), rainbow trout (*Oncorhynchus mykiss*) (7) and gilthead sea bream (*Sparus aurata*) (8), it was found that the mRNA levels of *npy*, *agrp* and *orexin* in brain regions increased after fasting. In addition to alterations in mRNA expression, studies have also documented neuronal changes associated with the perception of fish starvation following periods of fasting. For instance, fasting was found to increase number of NPY immunoreactive neurons in the hypothalamus and posterior tubercle nucleus in zebrafish (9). In tilapia (*Oreochromis mossambicus*), it was found that fasting enhanced the secretory activity of NPY neurons in the entopeduncular nucleus (NE) and preoptic nucleus (10). The findings from studies investigating the impact of fasting on POMC changes in fish are inconclusive. While fasting led to a decrease in *pomc* mRNA levels in brain regions of rainbow trout and zebrafish (11, 12), it resulted in an increase in *pomc* mRNA levels in flounder (*Paralichthys olivaceus*) (13). Conversely, fasting had no effect on *pomc* mRNA levels in goldfish (*Carassius auratus*) (14).

Lipid is the main nutrient involved in the maintenance of metabolic homeostasis in fish (15). In mammals, specific neurons in the hypothalamus have been shown to sense levels of long-chain fatty acids in the bloodstream for the regulation of energy homeostasis. These neurons exert their effects through various mechanisms: i) inhibiting carnitine palmitoyltransferase 1 (CPT1) to prevent the entry of fatty acid-coenzyme A into mitochondria for β-oxidation; ii) activating protein kinase C (PKC); iii) promoting electron leakage and subsequent mitochondrial ROS production; iv) increasing lipoprotein lipase (LPL) activity; v) regulating transcription factors involved in lipid metabolism, such as peroxisome proliferator-activated receptor α (PPARα) or sterol regulatory element-binding protein-c1 (SREBPC1), by binding to fatty acid transporters like FAT/CD36 (16–21). It has also been confirmed in fish that there is a fatty acid sensing system in the hypothalamus (22). The current research on the fatty acid sensing system in fish has primarily utilized methods such as high-fat diet feeding, intraperitoneal and intracerebral injection of fatty acids, and *in vitro* incubation of hypothalamic fragments with fatty acids (23–25). High-fat diets caused a decrease in the food intake of several fish species, including Senegalese sole (*Solea senegalensis*) (25), rainbow trout (24, 26), and grass carp (*Ctenopharyngodon idellus*) (27). After being fed a high-fat diet, rainbow trout exhibited significant inhibition of AMPK phosphorylation in the hypothalamus, while the AMPK pathway in the liver remained unaffected. Conversely, under high-fat diet stimulation, there was a significant increase in phosphorylation levels of protein kinase B (Akt) and mechanistic target of rapamycin complex (mTORC) in both the hypothalamus and liver (28). No studies have been reported investigating the fish fatty acid perception system through short-term fasting.

Animals are more inclined to catabolise ω-3 long-chain polyunsaturated fatty acids to meet their energy requirements during fasting (15). The long-chain polyunsaturated fatty acid eicosapentaenoic acid (EPA) is classified as an ω-3 polyunsaturated fatty acid, which holds significant physiological relevance in fish and plays a crucial role in the formation of biofilm structure and regulation of the immune system. After fasting, there was a significant decrease in EPA level throughout the entire body of chinook salmon (*Oncorhynchus tshawytscha*) (29), as well as a significant decrease in EPA level specifically within the liver of tiger puffer (*Takifugu rubripes*) (30). The response to EPA varies among different fish species. Intraperitoneal injection of EPA has been shown to activate the fatty acid sensing system in the hypothalamus of European sea bass (*Dicentrarchus labrax*) (31), but fails to activate this same system in the hypothalamus of Senegalese sole (25).

The tilapia is a significant economic fish species. Our previous research has shown that short-term fasting influences the responses of neuropeptides in the tilapia’s central nervous system and activates its glucose-sensing mechanism in the liver. Both lipid and glucose are critical nutrients (32). Nevertheless, it remains unclear whether short-term fasting affects the fatty acid sensing system in fish. Consequently, the present study utilized tilapia as an experimental model to investigate the effects of short-term fasting on serum fatty acid metabolism, with the objective of elucidating the relationship between short-term fasting and the fatty acid sensing system in fish. Moreover, this research aimed to provide insights into the potential mechanism by which the ω-3 long-chain polyunsaturated fatty acid EPA regulates feeding behavior and metabolism in tilapia.

## 2 Materials and methods

### 2.1 Animals

Juvenile male Nile tilapia (*Oreochromis niloticus*) weighing between 30 and 40 grams were sourced from Huadu Farm in Guangdong, China. The fish were housed in 150-L tanks at a density of 12 individuals per tank, maintained at a temperature of 28–30 °C under a light regime of 12 hours on and 12 hours off. Prior to the start of the experiment, the tilapia underwent acclimation for a minimum duration of two weeks and were fed twice daily until satiety was reached, with feeding times set at 8:00 a.m. and 5:00 p.m. Before dissection, all fish were anesthetized using MS222 (0.05%, Sigma; dissolved in water at a concentration of 0.1 g/L) or eugenol. All animal experiments adhered to the guidelines and received approval from the Animal Care and Use Committee at Sun Yat-sen University.

### 2.2 Experimental regimen

#### 2.2.1 Fasting experiment

Tilapia were randomly assigned to six control groups and four fasting groups, each consisting of 12 organisms. The control groups received full feedings for 30 Min at 8:00 AM and 5:00 PM. In contrast, the fasting groups were given full feedings for 30 Min at 8:00 AM, followed by a 24-hour fasting period. In the experiment, the starting point for fasting was 8:30 AM, which was defined as 0 h. In the control group, samples were collected at 0 and 4.5 h, with a total of 12 fish being sacrificed after being anesthetized with MS222 at each time point. In contrast, both the control and fasting groups were sampled at 9, 13.5, 18, and 24 h, with a total of 12 fish per group per time point being sacrificed following anesthesia using MS222. The sampling times were determined based on the outcomes of our previous study, which indicated significant alterations in the gastrointestinal contents of tilapia from the fasting group at these specific sampling intervals (32).

#### 2.2.2 Intraperitoneal injection experiment

A total of 180 tilapia (40–50 g) were randomly assigned to 15 tanks with 12 fish per tank. All tilapia were fasted for 24 h, anesthetized with an appropriate amount of eugenol, and intraperitoneally injected. 5 μL of EPA (Sigma) stock solution was dissolved into 917 μL of β-cyclodextrin solution (Sigma), which was configured into a 17 mM fatty acid master batch for storage, and diluted with PBS before each injection.

The experimental procedure for assessing the impact of intraperitoneal injection of EPA on the food intake and cumulative food intake of tilapia is as follows: three tanks of tilapia were injected with PBS, and the other three tanks were injected with EPA (300 μg/kg BW). The injection volume of each tilapia was 100 μL. After the injection was completed, the tilapia was placed back to the original fish tank for 30 Min (0 h), and fed after 1, 6 and 24 h of injection, respectively. The feeding time was 30 Min each time, and the food intake was counted. The food intake amounts refer to the weights of the feed consumed by the tilapia at 1 h, 6 h, and 24 h, respectively. The method for calculating cumulative food intake is as follows: 1 h cumulative food intake = 1 h food intake; 1–6 h cumulative food intake = 1 h food intake + 6 h food intake; and 1–24 h cumulative food intake = 1 h food intake + 6 h food intake + 24 h food intake.

Experiment on sampling tilapia after intraperitoneal injection of EPA. Five tanks of tilapia were injected with PBS solution, and four tanks of tilapia were injected with EPA solution (300 μg/kg BW). The EPA dose was selected on the basis of previous studies carried out in European sea bass (*Dicentrarchus labrax*) (31) and Senegalese sole (*Solea senegalensis*) (25). The PBS group was sampled at 0, 1, 3, 6 and 24 h after injection, and the EPA group was sampled at 1, 3, 6 and 24 h after injection. One tank of fish was taken at each time point in PBS group and EPA group.

#### 2.2.3 Sampling procedure

1 mL of blood was collected from the tilapia using a 1 mL medical syringe, and the fish were then euthanized by decapitation. For RNA extraction, hypothalamus and liver samples were flash-frozen in liquid nitrogen, while hypothalamus and liver samples intended for Western blot analysis were homogenized in ice-cold RIPA lysis buffer on the day of sampling (Beyotime, China).

### 2.3 RNA extraction and real-time quantitative PCR

The frozen hypothalamus and liver were homogenized in TRIzol (Omega, Norcross, GA, USA) and total RNA was extracted according to the following protocol (33). A total of 1 µg of total RNA was reverse transcribed into cDNA using the RevertAid First Strand cDNA Synthesis Kit (Life Technology, Carlsbad, USA), following the cycling protocol provided by the manufacturer. Quantitative real-time polymerase chain reaction (qPCR) was set up in 10 µL reaction mixtures containing 5 µL of 2× SYBR Green qPCR Master Mix, 1 µL of a 100 ng/µL cDNA dilution solution, 0.3 µL of a 10 µM sense primer, 0.3 µL of a 10 µM anti-sense primer, and 3.4 µL of ddH_2_O (Bimake, Houston, TX, USA). The cycling protocol of qPCR was conducted according to the manufacturer’s instructions. All samples were run in duplicate, and β-actin was used as the internal normalization control. The sequences of the oligonucleotide primers employed in this study are detailed in **Table 1**. The 2^(−ΔΔCT) method was utilized to ascertain the relative mRNA expression levels of the target gene in comparison to the reference gene (34).

**Table 1:** Sequences of primers used in quantitative real-time PCR.

Gene	Forward primer sequence (5 '-3')	Reverse primer sequence (5 '-3')	Sequence source	Product size
*Neuropeptide Y* (*npy*)	ACAAGACAGAGGTATGGGAAGA	GGCAGCATCACCACATTG	XM_003448854.5	130bp
*Proopiomelanocortin* (*pomc*)	GGCAAACCAGCAGGACTT	AGCCTGAAAGGGAAAACG	XM_005454720.4	186bp
*Peroxisome Proliferator-Activated Receptor Alpha* (*pparα*)	GTTCCTCAAGAGTCTCCGCC	AAAGAGCTAGGTCGCTGTCG	ENSONIG00000016715	122bp
*Peroxisome Proliferator-Activated Receptor Alpha* (*kir10*)	CCAAGTCCTGTCACTTTGGGA	GTCATCTTTTCAGCGCAGGC	ENSONIG00000015152	282bp
*Hydroxyacyl-CoA Dehydrogenase* (*hadh*)	TTGGTATGAAACTCGGCGCT	CAAGTTTGCCCTCTGCAACC	ENSONIG00000017504	171bp
*Lipoprotein Lipase* (*lpl*)	GATACACGGCTGGACGGT	TCGGGTAGTGCTGATTGG	NM_001279753.1	134bp
*sterol regulatory element-binding protein-c1* (*srebpc1*)	ATTCAGCCAGTGGCCATTCA	CAGGGGTCAGGCTTCAAAGT	XM_005457771.4	185bp
*Fatty Acid Synthase* (*fas*)	CCAGACTTCAGAGACTCCATTC	TGCGTGAACTGTGTCTTCAA	XM_013276808.2	117bp
*Acetyl-CoA Carboxylase Alpha* (*accα*)	GCTTCCAGCACAATAGTATCG	CACTGTTCCTGAGATTGACATC	XM_019363864.1	138bp
*Palmitoyltransferase 1 Alpha* (*cpt1α*)	TTTCCAGGCCTCCTTACCCA	TTGTACTGCTCATTGTCCAGCAGA	XM_003440552.5	102bp
*β-actin*	ACCTTCTACAACGAGCTGAGAG	GCCTGGATGGCAACGTACA	AB037865	149bp

### 2.4 Transcriptome analysis by RNA sequencing

Hypothalamus and liver tissues were collected from tilapia in the control group at 0, 4.5, 9, 13.5, 18, and 24 h as well as from the fasting group at 9, 13.5, 18, and 24 h for total RNA extraction (n=5). The experimental protocol was conducted according to Yu et al (32). The purity, concentration, and integrity of the RNA samples were evaluated using a Qubit 2.0 fluorometer (Thermo Fisher, USA), a NanoDrop 2000 spectrophotometer (Thermo Fisher, USA), and an Agilent 2100 bioanalyzer (Agilent, USA), respectively. cDNA libraries were constructed and subsequently subjected to high-throughput sequencing using a HiSeq4000 sequencer (Illumina, USA). The reference genome and gene annotation files employed in the analysis were obtained from the genome website (https://asia.ensembl.org/Oreochromis_niloticus/Info/Index). An index of the reference genome was constructed using Hisat2, and paired-end clean reads were aligned to the reference genome. The mapping reads of each sample were assembled using StringTie 1.3.3 in a reference-based approach. The FeatureCounts 1.50 software was employed to enumerate the number of reads that were aligned to each gene.

### 2.5 Measurements of biochemical parameters

Triglyceride (TG), high-density lipoprotein cholesterol (HDL-C), low-density lipoprotein cholesterol (LDL-C), very low-density lipoprotein (VLDL) and total cholesterol (T-CHO) in the serum were quantified using commercial kits (Jiancheng Biotech Co., China) following the manufacturer’s instructions.

### 2.6 Western blot analysis

The protein concentration in the hypothalamus and liver samples was quantified using a BCA protein assay kit (Beyotime, China), followed by western blot analysis. The experimental protocol was conducted according to Zhao et al. (35). The antibody against rabbit phospho-AMPKα (Thr172) (cell signaling technology, USA), antibody against rabbit AMPKα (cell signaling technology, USA), antibody against rabbit Akt (pan) (C67E7) (#4691, cell signaling technology, USA), and antibody against rabbit Phospho-Akt (Ser473) (D9E) (#4060, cell signaling technology, USA), and antibody against mouse β-actin (proteintech, USA) were employed. The secondary antibodies used were either an HRP-conjugated goat anti-rabbit IgG antibody (ProteinTech, USA) or an HRP-conjugated goat anti-mouse IgG antibody (ProteinTech, USA), both of which were diluted to a concentration of 1:5000. Gray intensity analysis was conducted using Image J 1.45 software (NIH, Bethesda, USA).

### 2.7 Immunofluorescence

Intact tilapia brains were obtained and fixed in 4% paraformaldehyde at 4°C for 18 hours. Thereafter, the brains were transferred to a 30% sucrose solution at 4°C until the brain sank to the bottom. The brain was embedded using OCT (Sakura), sectioned at a thickness of 10 μm, and the slices were dried at 37°C for four hours. Antigen repair was performed using sodium citrate thermal repair solution (pH=6.0) in a microwave oven at 800 W for five minutes. Subsequently, the slices were washed three times with PBS containing 0.3% hydrogen peroxide for 10 minutes each time. The sections were then blocked with 1% Triton×100 goat serum (Boster, China) at room temperature for 1 hour. Anti-NPY antibody (N9528, Sigma) or anti-POMC antibody (ab210605, Abcam) was diluted 1:1000 in goat serum to prepare the primary antibody, shake off the blocking solution, add the appropriate amount of primary antibody dropwise, and put into a wet box at 4 °C overnight. Shake off the primary antibody and soak in PBS 3 times, each time for 5 Min. Goat anti-rabbit IgG-Alexa Fluor^®^ 488 (Abcam) was diluted in goat serum at 1:1000, and an appropriate amount of secondary antibody was added dropwise to the suspension on the section, and then put into a wet box reacted at room temperature for 1 h in dark. Soaked in PBS 3 times, each time for 5 Min. Use the anti-fluorescent anti-DAPI solution to quench the sealing solution (DAPI) and then put into a wet box and react for 1 hour. The samples were covered with DAPI-containing anti-fluorescence quenching sealing solution (Beyotime, China), sealed and stored at -20 °C. Images were captured separately using a fluorescent microscope (Eclipse Ni-E, Nikon, Japan) equipped with appropriate excitation filters for DAPI and Alexa Fluor 488. The NIS-Elements Viewer 5.12 software (Nikon, Japan) was then utilized to overlay the two images.

### 2.8 Metabolite extraction and LC-MS/MS analyses

Transcriptomic profiling was performed on serum samples collected from fasted and control tilapia, with a focus on changes in metabolites at 0, 9, 13.5, 18, and 24 h post-fasting. The blood sample was processed according to Yu et al (32). The quality control (QC) sample was prepared through the combination of equal aliquots of the supernatants derived from each blood sample. Liquid chromatography-tandem mass spectrometry (LC-MS/MS) analyses were performed using an ultra-high-performance liquid chromatography (UHPLC) system (Vanquish, Thermo Fisher Scientific) with a UPLC BEH Amide column (2.1 mm × 100 mm, 1.7 μm) coupled to an Orbitrap MS (Thermo Scientific). The QE HFX mass spectrometer was employed due to its capacity to obtain MS/MS spectra through information-dependent acquisition (IDA) in the context of control by the acquisition software (Xcalibur, Thermo). The conditions for the ESI source were established as follows: sheath gas flow rate as 30 Arb, Aux gas flow rate as 25 Arb, capillary temperature 350 °C, full MS resolution as 60000, MS/MS resolution as 7500, collision energy as 10/30/60 in NCE mode, spray voltage of 3.6 kV (positive) or -3.2 kV (negative), respectively. The raw data underwent conversion to the mzXML format via ProteoWizard, following which processing was conducted using an in-house program. This was developed with the use of R and is based on XCMS for peak detection, extraction, alignment, and integration purposes. Subsequently, an in-house MS2 database (BiotreeDB) was employed for metabolite annotation. The threshold for annotation was set at 0.3. The differential changed metabolites were identified according to two criteria: variable influence on projection (VIP > 1) and p-value < 0.05.

### 2.9 Statistical analysis

The quantitative data were presented as mean ± SEM. The statistical analyses were conducted using the IBM SPSS Statistics software, version 24 (IBM Corp., Armonk, NY, USA). For normally distributed data, a Student’s t-test was used to compare two groups, while a one-way ANOVA was employed for comparisons among multiple groups, with Duncan’s method utilized for multiple comparisons. Conversely, non-parametric tests were utilized in cases where the data did not meet the criteria for normal distribution, and the Mann-Whitney U test was applied for comparisons between two groups, and multiple group comparisons were performed using the Kruskal-Wallis one-way ANOVA, followed by a stepwise reduction of multiple comparison methods.

## 3 Results

### 3.1 The serum fatty acid levels in tilapia are altered by short-term fasting

The impact of short-term fasting on serum fatty acid levels in tilapia is illustrated in **Table 2**. The results suggested that the levels of long-chain saturated and long-chain monounsaturated fatty acids in tilapia serum were significantly increased following short-term fasting, while the levels of long-chain polyunsaturated fatty acids were significantly reduced. After fasting for 18–24 h, the short-chain fatty acid 3-Methylthiopropionic acid in tilapia serum decreased significantly. During fasting for 9–24 h, long-chain saturated fatty acids 2-Hydroxymyristic acid, Hexadecanedioic acid, Heptadecanoic acid, Stearic acid, 2-Hydroxystearic acid, Arachidic acid and long-chain monounsaturated fatty acids (9xi,10xi,12xi)-9,10-Dihydroxy-12-octadecenoic acid, Petroselinic acid, 11Z-Eicosenoic acid were significantly up-regulated. Long-chain saturated fatty acid 3-Methyl-5-pentyl-2-furanundecanoic acid was significantly up-regulated after fasting for 9 and 13.5 h, but significantly down-regulated after fasting for 18 and 24 h. The long-chain polyunsaturated fatty acids Arachidonic acid and Eicosapentaenoic acid (EPA) were up-regulated after fasting for 9 h and significantly down-regulated after fasting for 13.5,18 and 24 h (**Table 2**).

**Table 2:** Different metabolites of fatty acids in serum of tilapia during fasting for 24 h.

Compound	Carbon chain length	Number of double bonds	Log_2_(Fold-change)			
			F9/0	F13.5/0	F18/0	F24/0
3-Methylthiopropionicacid	4	0	**0.99**	**0.46**	**-0.71**	**-1.94**
2-Hydroxymyristicacid	14	0	**0.22**	**1.03**	**0.7**	**1.27**
Hexadecanedioicacid	16	0	**0.23**	**0.26**	**0.32**	**1.22**
Heptadecanoicacid	17	0	**0.52**	**1.11**	**0.59**	**1.81**
Stearicacid	18	0	**0.68**	**1.25**	**0.99**	**1.85**
2-Hydroxystearicacid	18	0	**0.42**	**1.59**	**1.29**	**2.2**
Arachidicacid	20	0	**1.14**	**1.25**	**0.65**	**0.87**
3-Methyl-5-pentyl-2-furanundecanoicacid	21	0	**2.03**	**0.89**	**-0.59**	**-0.74**
(9xi,10xi,12xi)-9,10-Dihydroxy-12-octadecenoicacid	18	1	**-0.14**	**0.1**	**0.32**	**1.57**
Petroselinicacid	18	1	**0.73**	**0.67**	**0.15**	**1.23**
11Z-Eicosenoicacid	20	1	**1.23**	**1.47**	**0.82**	**1.76**
Arachidonicacid	20	4	**0.44**	**-0.3**	**-1.15**	**-0.72**
Eicosapentaenoicacid	20	5	**0.25**	**-0.53**	**-1.49**	**-0.82**

F9/0, F13.5/0, F18/0, and F24/0 represent the logarithm of the fold change between the 9-hour, 13.5-hour, 18-hour, and 24-hour fasting groups and the control group (0-hour fasting group), respectively. A positive logarithmic fold change reflects upregulation, while a negative logarithmic fold change indicates downregulation. The magnitude of the absolute value of the numerical data correlates with the extent of the change. Red is used to signify an increase, whereas blue represents a decrease.

The bold values indicate logarithm results of the fold change of corresponding groups. The calculation formula is: log2(treat group/control group).

### 3.2 Fasting for 24 h activates fatty acid-sensing system in hypothalamus and liver

**Figure 1:**
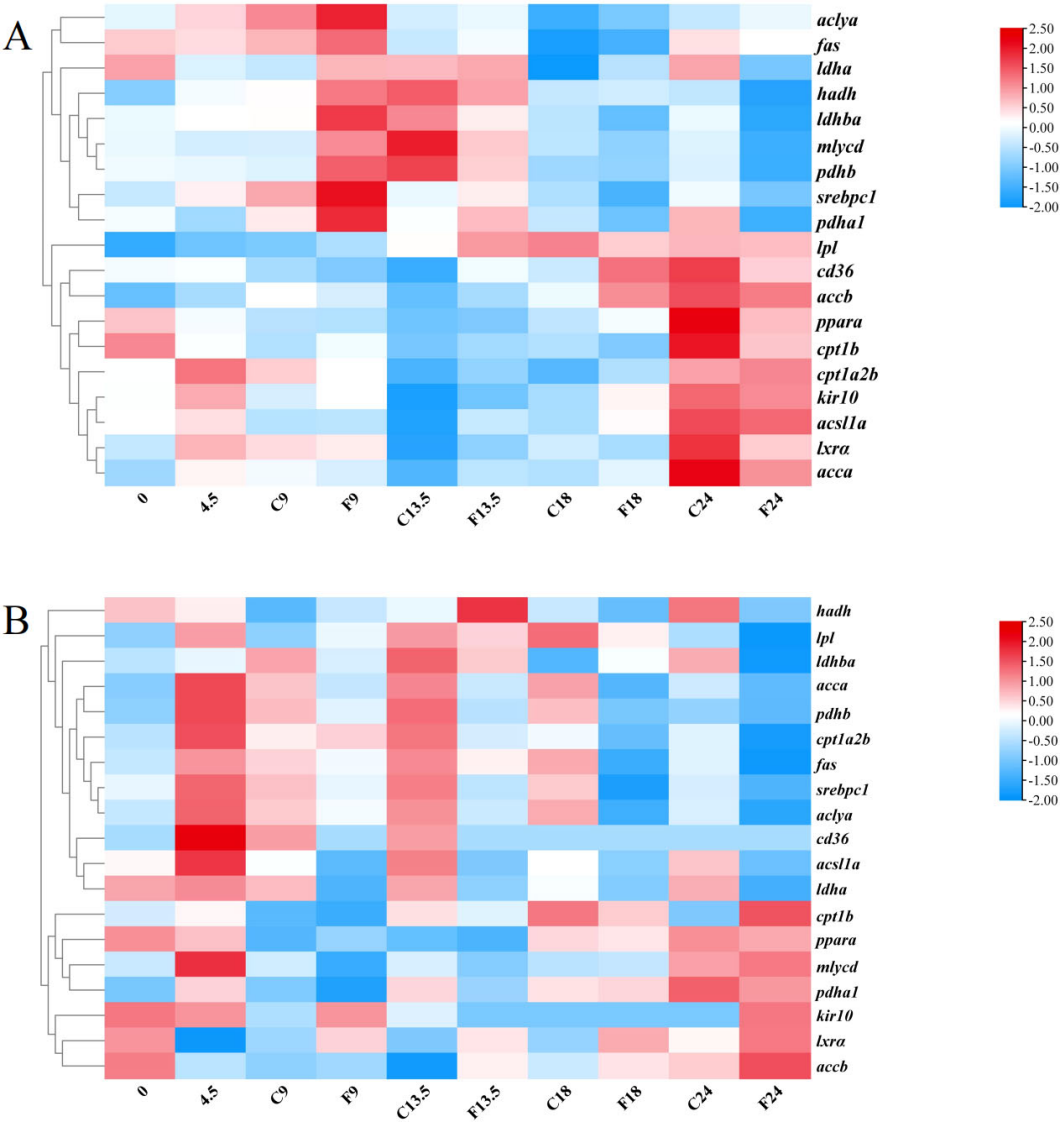
Clustering Analysis of fatty acid-sensing genes in the hypothalamus and liver of tilapia. **(A)** Analysis of fatty acid sensing related genes in the hypothalamus. **(B)** Analysis of fatty acid sensing related genes in the liver. Each row represented the temporal expression pattern of a single gene and each column represented the average of the five samples in the group.0, 4.5, C9, C13.5, C18, and C24 represent the control groups of tilapia at 0, 4.5, 9, 13.5, 18, and 24 hours, respectively. F9, F13.5, F18, and F24 represent the fasting groups of tilapia at 9, 13.5, 18, and 24 hours, respectively. The expression levels of genes were represented by colored tags, with red representing higher levels of expression and blue representing lower levels of expression.

We conducted transcriptome sequencing on the hypothalamus and liver of tilapia from the control group (0, 4.5, 9, 13.5, 18, and 24 h) and the fasting group (9, 13.5, 18, and 24 h). In the present research, we focused on fatty acids sensing systems mediated by fatty acid metabolism, CD36, mitochondrial activity, protein kinase C and lipoprotein lipase (**Figure 1**). Compared with the control group, the mRNA expression levels of ATP-citrate lyase a (*aclya*), fatty acid synthase (*fas*), malonyl-CoA decarboxylase (*mlycd*), pyruvate dehydrogenase E1 subunit beta (*pdhβ*) and pyruvate dehydrogenase E1 subunit alpha 1 (*pdhα1*), lactate dehydrogenase A4 (*ldha*), lactate dehydrogenase Ba (*ldhba*) in the hypothalamic fatty acid metabolism perception pathway of tilapia in the fasting group showed a consistent trend, which increased significantly after fasting for 9 h and decreased significantly after 24 h. While the mRNA levels of accα, Acetyl-CoA carboxylase beta (*accβ*) and Acyl-CoA synthetase long-chain family member 1a (*acsl1a*) in the fasting group were not significantly different from those in the control group after fasting for 9 h. After fasting for 24 h, there was no significant differences compared with the control group, but it was significantly up-regulated compared with 0 h. In the perception mechanism regulated by CD36, the mRNA level of *srebpc1* in the hypothalamus of tilapia in the fasting group was significantly higher than that in the control group and the 0 h group after fasting for 9 h, and returned to the level of 0 h after fasting for 24 h. Compared with the control group, the mRNA levels of *cd36*, *lxrα* and *pparα* in the hypothalamus of tilapia in the fasting group did not change significantly at 9 h and 24 h. Compared with the control group, the mRNA level of *hadh* in the hypothalamic mitochondrial activity perception mechanism of tilapia in the fasting group was significantly up-regulated after fasting for 9 h and significantly down-regulated after fasting for 24 h. There was no significant change in *cpt1a2b* and *cpt1b* mRNA level after fasting for 9 h, but it was down-regulated after fasting for 24 h. There were no significant difference in mRNA levels of ATP-sensitive inward rectifier potassium channel 10 (*kir10*) in the activated protein kinase C perception mechanism and *lpl* in lipoprotein perception mechanism in hypothalamus between the short-term fasting group and the control group (**Figure 1A**).

The liver transcriptome data showed that compared with the control group, the mRNA levels of *aclya*, *fas*, *accα*, *acsl1 a*, *ldha* and *ldhba* in the fatty acid metabolism perception pathway of tilapia liver in the fasting group were significantly down-regulated after fasting, and the mRNA level of *accb* was significantly up-regulated after fasting for 24 h. While the mRNA levels of *mlycd*, *pdhb* and *pdha1* did not change significantly during fasting. In the perception mechanism regulated by CD36, the *lxrα* mRNA level in the liver of tilapia in the fasting group was significantly higher than that in the control group during 9–24 h of fasting, and the *cd36*, *pparα*, and *srebpc1* mRNA levels did not change significantly. The *cpt1b* mRNA level in the liver mitochondrial activity sensing mechanism of tilapia in the fasting group was significantly higher than that in the control group after 24 h of fasting, and the *cpt1a2b* and *hadh* were significantly lower than those in the control group after 24 h of fasting. The *kir10* mRNA level in the liver-activated protein kinase C sensing mechanism of tilapia in the fasting group was significantly higher than that in the control group after 24 h of fasting, and the *lpl* mRNA level in the lipoprotein lipase sensing mechanism was significantly lower than that in the control group after 24 h of fasting (**Figure 1B**).

### 3.3 Fasting for 24 hours changed POMC and NPY protein levels in tilapia brain

**Figure 2:**
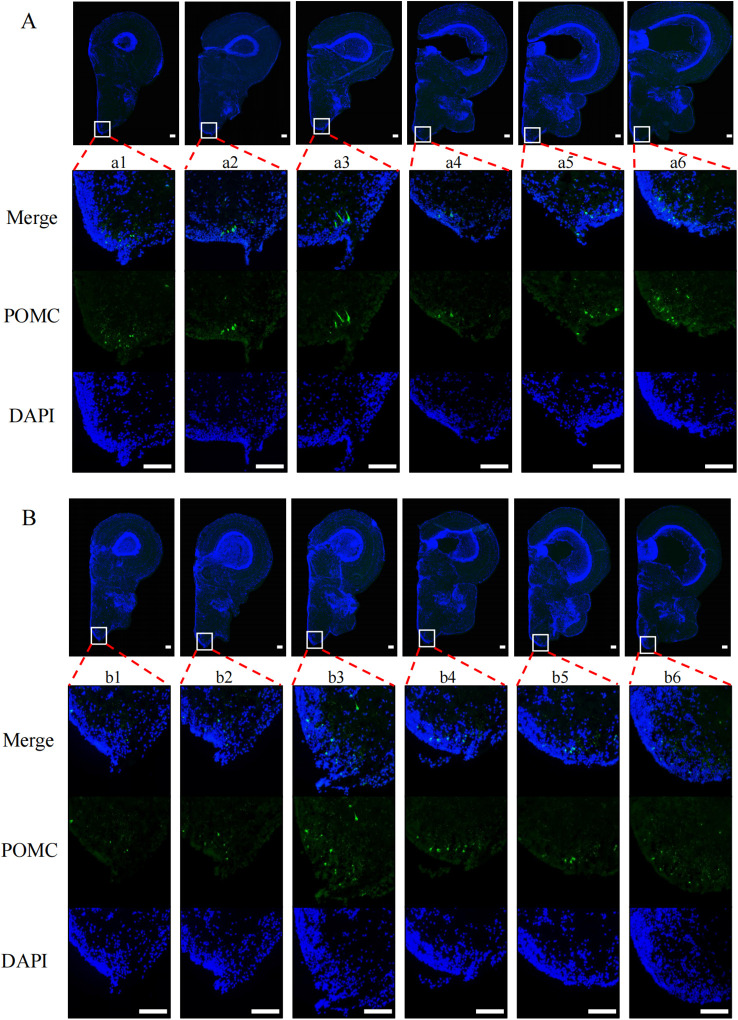
Immunofluorescence photomicrographs of POMC (green) in midbrain region of tilapia under different nutritional states. **(A)** Results from the midbrain of satiated tilapia, **(B)** Results from the midbrain of tilapia after 24 hours of fasting. Merge represents that the resulting plots of POMC and DAPI overlap. POMC and DAPI refer to the resulting plot of POMC and DAPI, respectively. The scale bar measures 100 μm.

**Figure 3:**
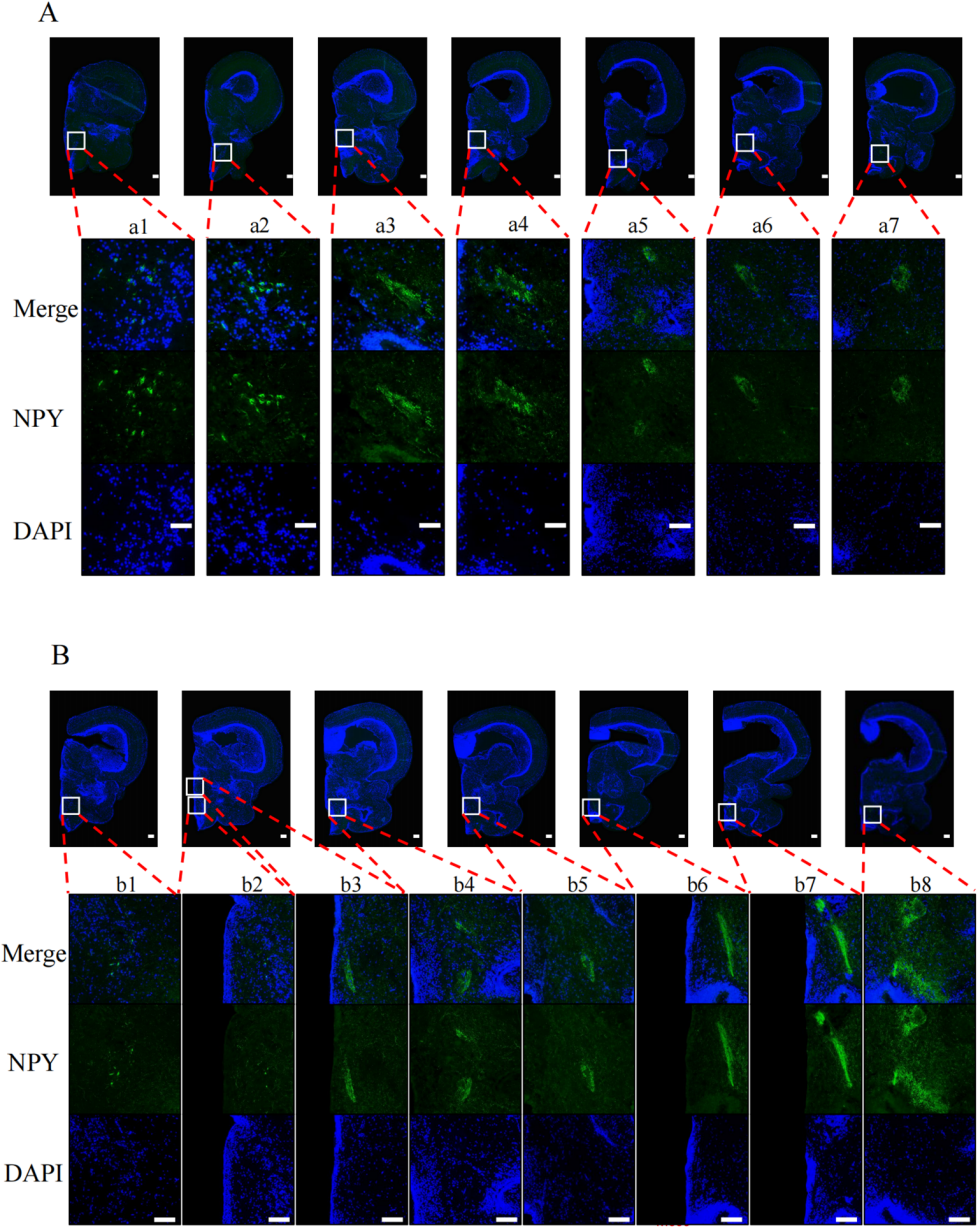
Immunofluorescence photomicrographs of NPY (green) in midbrain of tilapia under different nutritional states. **(A)** Results from the midbrain of satiated tilapia, **(B)** Results from the midbrain of tilapia after 24 hours of fasting. Merge represents that the resulting plots of POMC and DAPI overlap. POMC and DAPI refers to the resulting plot of POMC and DAPI, respectively. The scale bar measures 100 μm.

To explore the effects of nutritional status on POMC and NPY protein levels in tilapia brain regions, we performed immunofluorescence experiments on tilapia brain regions after satiation and fasting for 24 h. After fasting for 24 h, the POMC signal (**Figures 2A, B**) in the nLT (**Figure 2**b2) of the fasting group was weaker than that of the satiation feeding group (**Figure 2**a3). The NPY signal (**Figures 3A, B**) in the nPrG region (**Figure 3**a6) of tilapia in the fasting group was stronger than that in the satiation feeding group (**Figure 3**b6).

### 3.4 EPA inhibits appetite through regulating *pomc*

**Figure 4:**
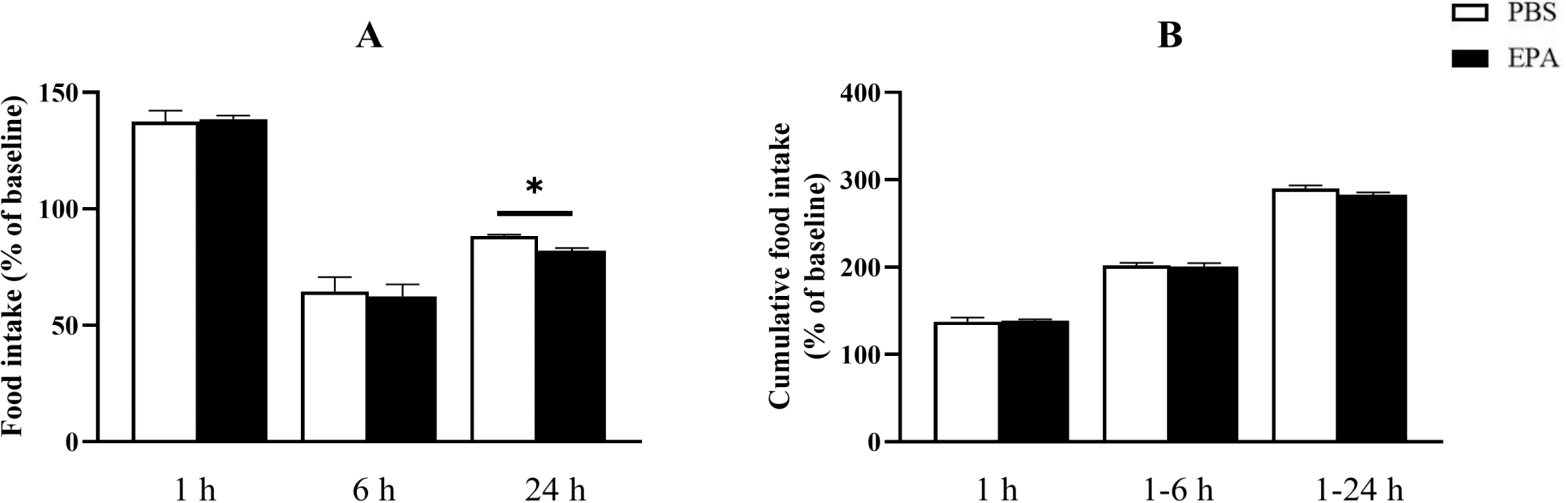
Effects of intraperitoneal administration of EPA on food intake in tilapia. **(A)** The results of food intake in tilapia following injection at 1, 6, and 24 h. **(B)** The results of cumulative food intake in tilapia following injection at 1, 6, and 24 (h) The method for calculating cumulative food intake is as follows: 1 h cumulative food intake = 1 h food intake; 1–6 h cumulative food intake = 1 h food intake + 6 h food intake; and 1–24 h cumulative food intake = 1 h food intake + 6 h food intake + 24 h food intake. Mean ± standard error, n=3. * means significant differences, *p<0.05.

**Figure 5:**
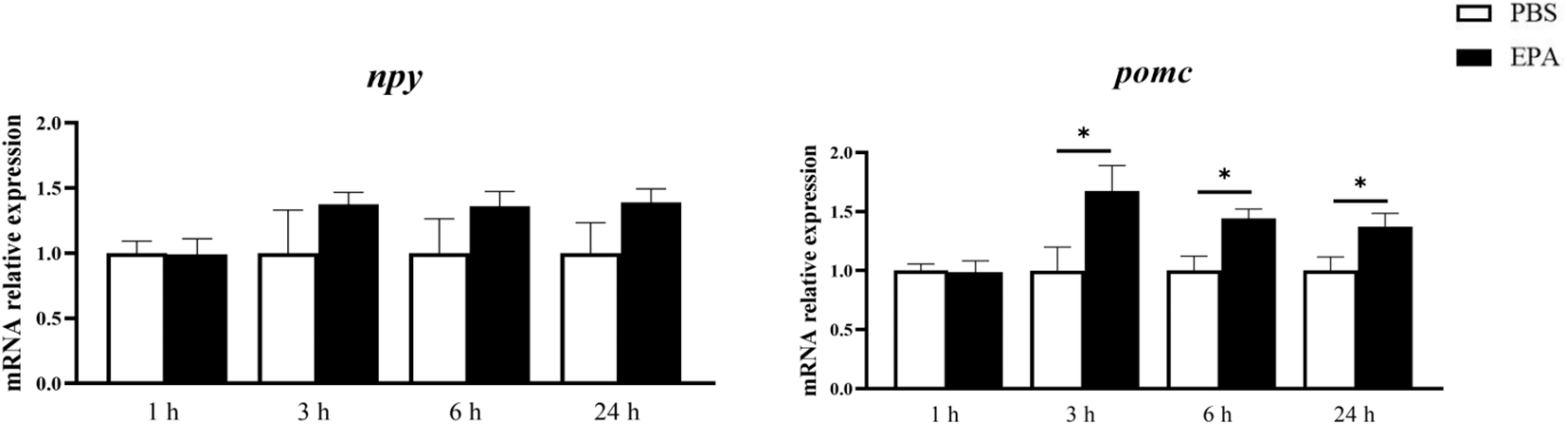
Effects of intraperitoneal administration of EPA on hypothalamus feeding-related genes in tilapia. *npy*, Neuropeptide Y; *pomc*, Proopiomelanocortin. 1 h, 3 h, 6 h, and 24 h represent the tilapia groups that were sampled at 1 hour, 3 hours, 6 hours, and 24 hours, respectively. Mean ± standard error, n=8. * means significant differences, *p<0.05.

In order to assess the impact of EPA on post-fasting appetite in tilapia, intraperitoneal administration of EPA was performed, and food intake as well as cumulative food intake were measured at 1, 6, and 24 hours post-injection. No significant effects on food intake or cumulative food intake were observed in tilapia at 1 hour and 6 hours following intraperitoneal EPA injection. However, after 24 hours, there was a notable decrease in tilapia’s food intake (**Figure 4**). Furthermore, the mRNA levels of the feeding-related gene *pomc* in the hypothalamus exhibited significant increases at 3, 6, and 24 hours after intraperitoneal EPA injection; however, no significant changes were observed in *npy* mRNA levels (**Figure 5**).

### 3.5 EPA activates fatty acid-sensing systems in the hypothalamus and liver

**Figure 6:**
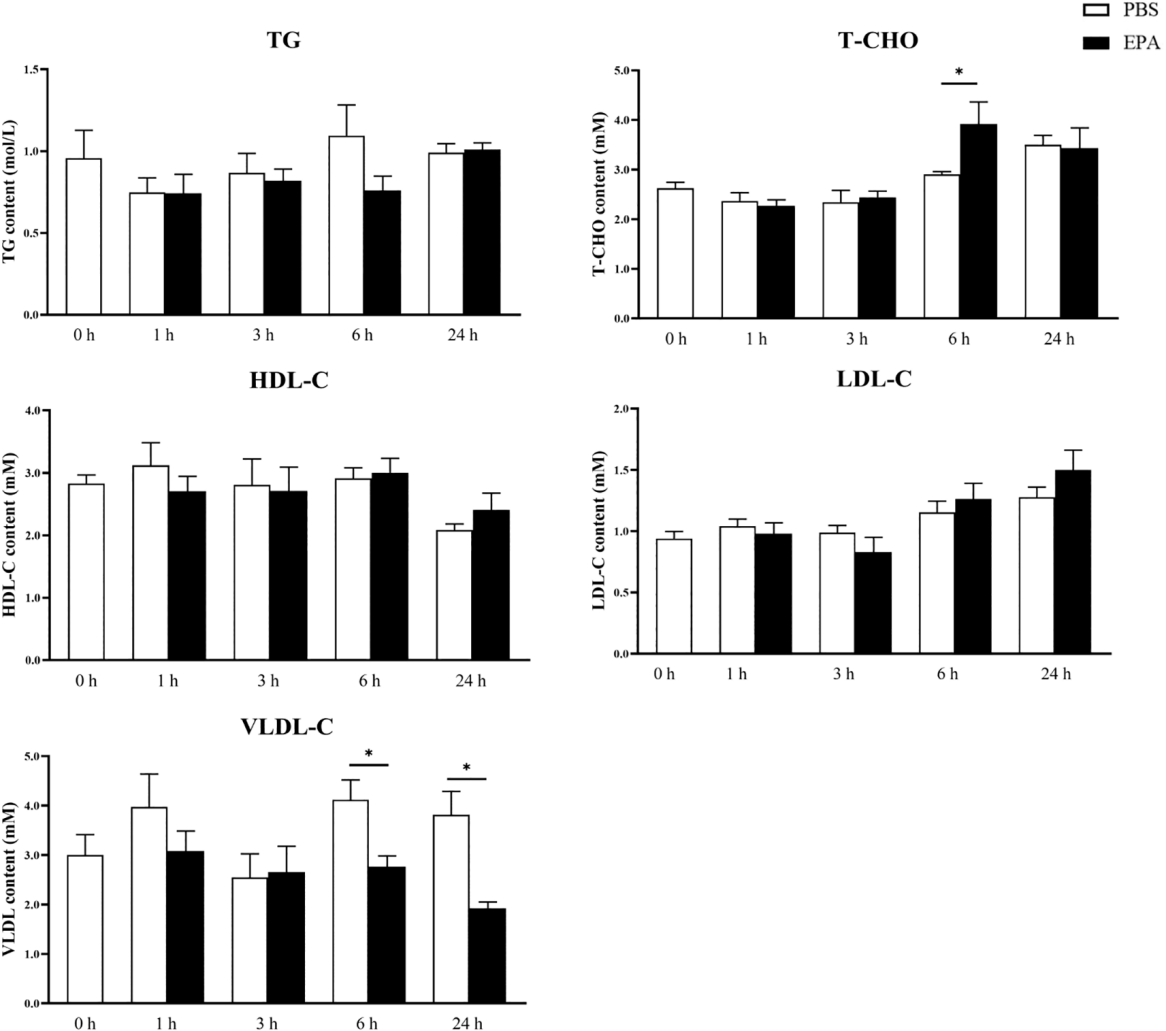
Effects of intraperitoneal administration EPA on serum physiological indexes of tilapia. TG, Triglyceride; T-CHO, Total cholesterol; HDL-C, High-density lipoprotein cholesterol; LDL-C, low-density lipoprotein cholesterol; VLDL, very low-density lipoprotein. 0 h, 1 h, 3 h, 6 h, and 24 h represent the tilapia groups that were sampled at 0 hour, 1 hour, 3 hours, 6 hours, and 24 hours, respectively. Mean ± standard error, n=5-8. * means significant differences, *p<0.05.

**Figure 7:**
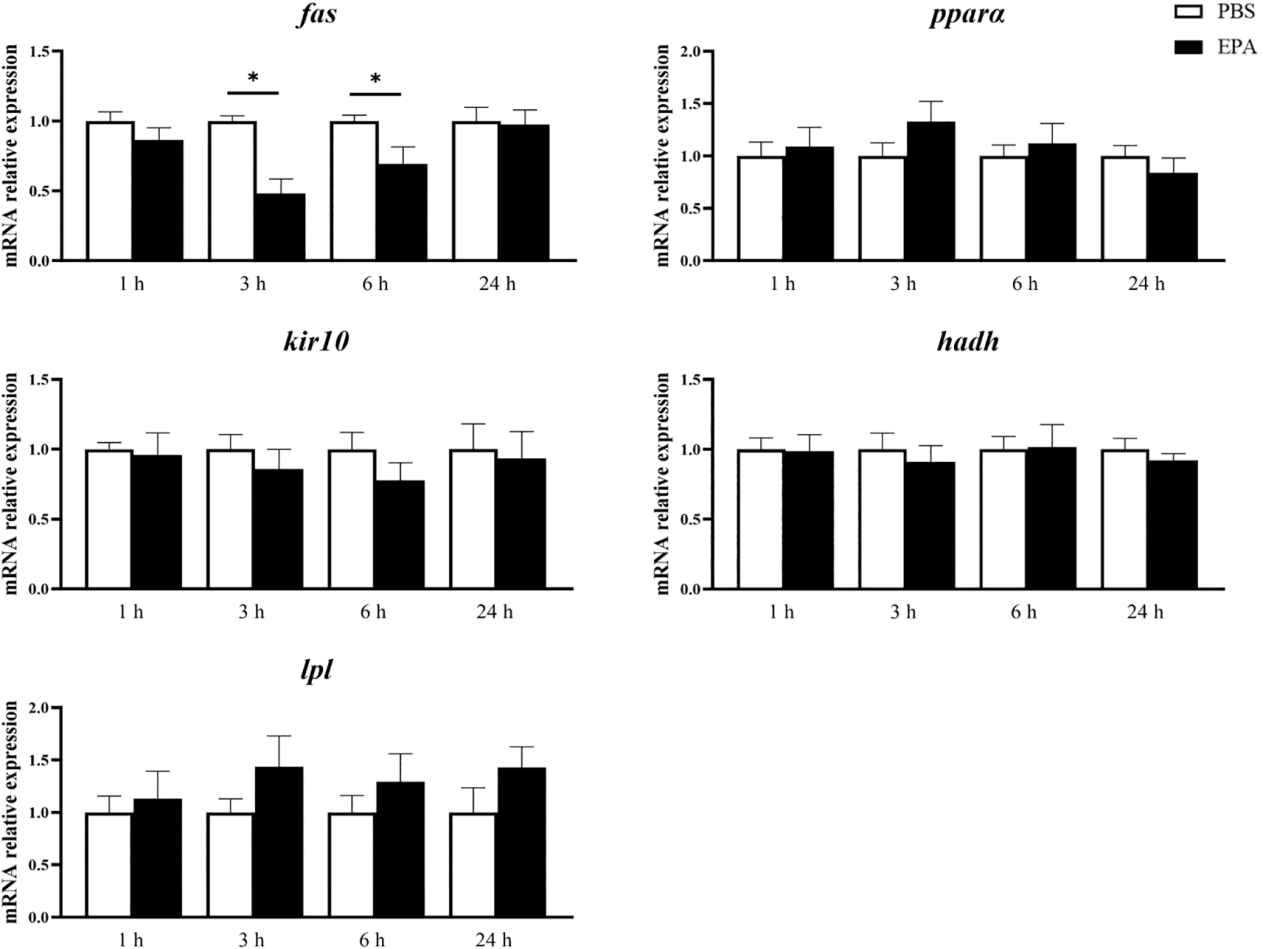
Effects of intraperitoneal administration of EPA on genes related to fatty acid sensing system in tilapia hypothalamus. *fas*, Fatty acid synthase; *pparα*, Peroxisome proliferator activated receptor α; *kir 10*, ATP-sensitive inward rectifier potassium channel 10; *hadh*, Hydroxyacyl-CoA dehydrogenase; *lpl*, Lipoprteinlipase. 1 h, 3 h, 6 h, and 24 h represent the tilapia groups that were sampled at 1 hour, 3 hours, 6 hours, and 24 hours, respectively. Mean ± standard error, n=7-8. * means significant differences, *p<0.05.

**Figure 8:**
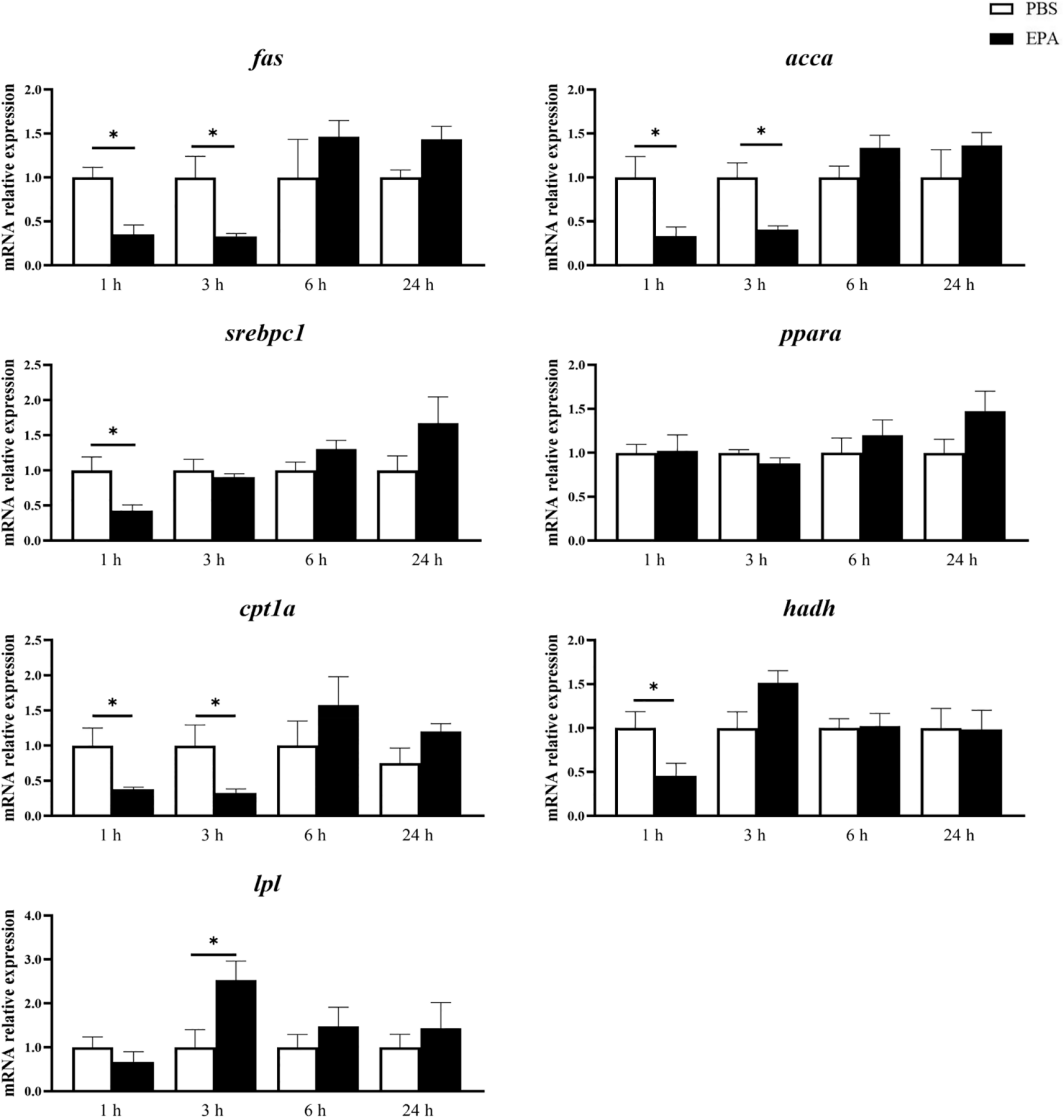
Effects of intraperitoneal administration of EPA on genes related to fatty acid sensing system in tilapia liver. *fas*, Fatty acid synthase; acc*α:* Acetyl-CoA carboxylase α*; srebpc1*, Sterol regulatory element-binding protein-c1; *pparα*, Peroxisome proliferator activated receptor α; cpt1α, Palmitoyltransferase 1 α; *hadh*, Hydroxyacyl-CoA dehydrogenase; *lpl*, Lipoprteinlipase; 1 h, 3 h, 6 h, and 24 h represent the tilapia groups that were sampled at 1 hour, 3 hours, 6 hours, and 24 hours, respectively. Mean ± standard error, n=7-8. * means significant differences, *p<0.05.

In order to investigate the impact of EPA on the fatty acid sensing system of tilapia, we administered intraperitoneal injections of EPA to fasted tilapia and analyzed changes in lipid-related metabolites in serum as well as key genes involved in the fatty acid sensing system of the hypothalamus and liver. Following a 6-hour injection period, there was a significant decrease in VLDL-C levels and a significant increase in T-CHO levels within the serum of tilapia. After 24 hours of intraperitoneal injection, there was also a notable reduction in VLDL-C levels within the serum of tilapia (**Figure 6**). Following the intraperitoneal administration of EPA, a significant reduction in *fas* mRNA levels was observed in the hypothalamus of tilapia at both 3 and 6 hours post-injection. Conversely, the levels of *pparα*, *hadh*, *kir10* and *lpl* mRNA remained largely unaltered (**Figure 7**). In the liver, the levels of *fas*, *accα* and *cpt1α* mRNA were significantly reduced after 1 and 3 hours, while the levels of *srebpc1* and *hadh* mRNA were significantly reduced after 1 hour. In contrast, the *lpl* mRNA level was significantly increased after 3 hours. No significant change was observed in the *pparα* mRNA level (**Figure 8**).

### 3.6 EPA affected AMPK and AKT signaling pathways in hypothalamus and liver

**Figure 9:**
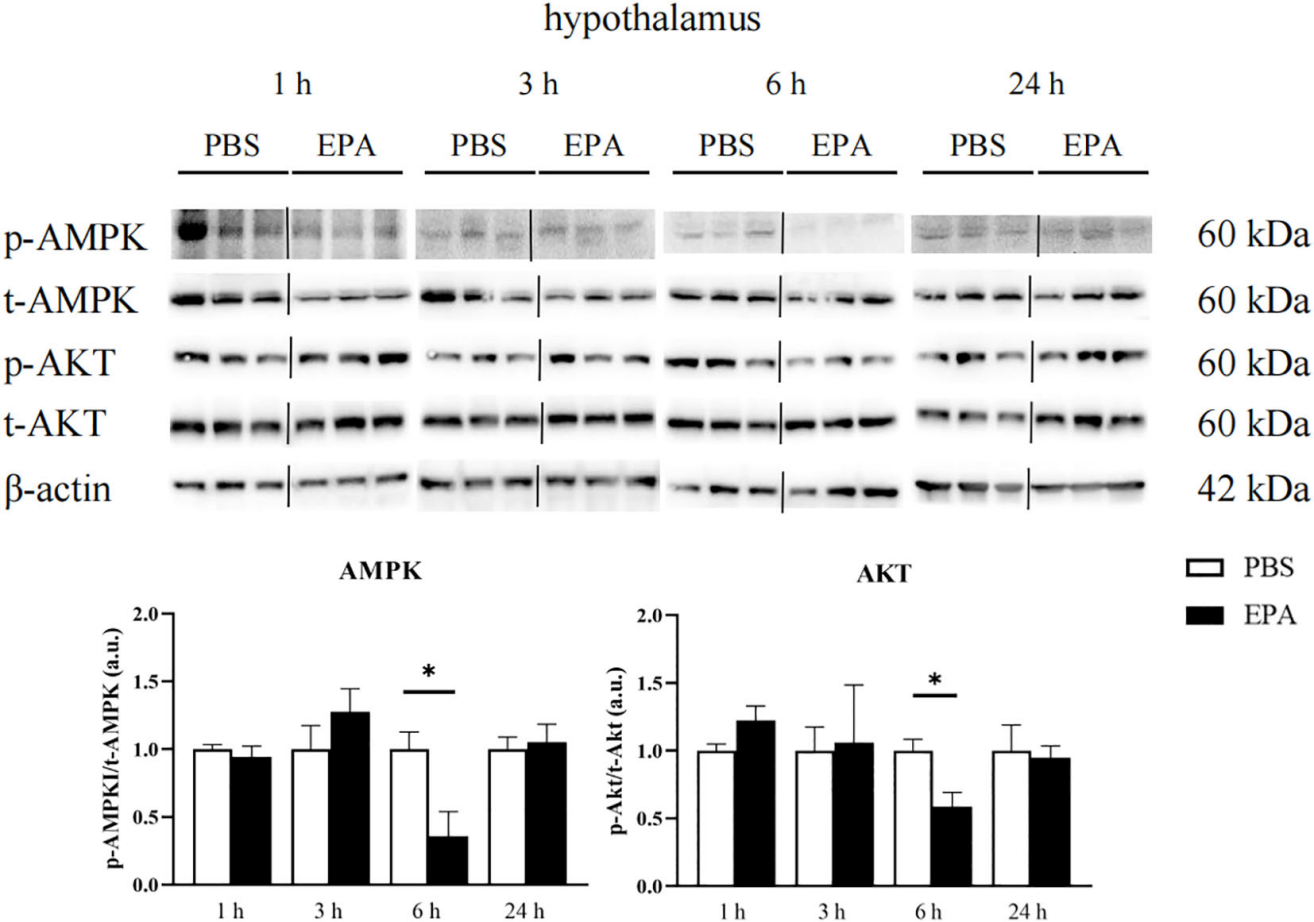
Effects of intraperitoneal administration of EPA on AMPK and AKT signaling pathways in tilapia hypothalamus. p-AMPK, phosphorylated AMPK protein; t-AMPK, total AMPK protein; p-AKT, phosphorylated AKT protein; t-AKT, total AKT protein; β-actin, β-actin protein. 1 h, 3 h, 6 h, and 24 h represent the tilapia groups that were sampled at 1 hour, 3 hours, 6 hours, and 24 hours, respectively. Mean ± standard error, n=3. * means significant differences, *p<0.05.

**Figure 10:**
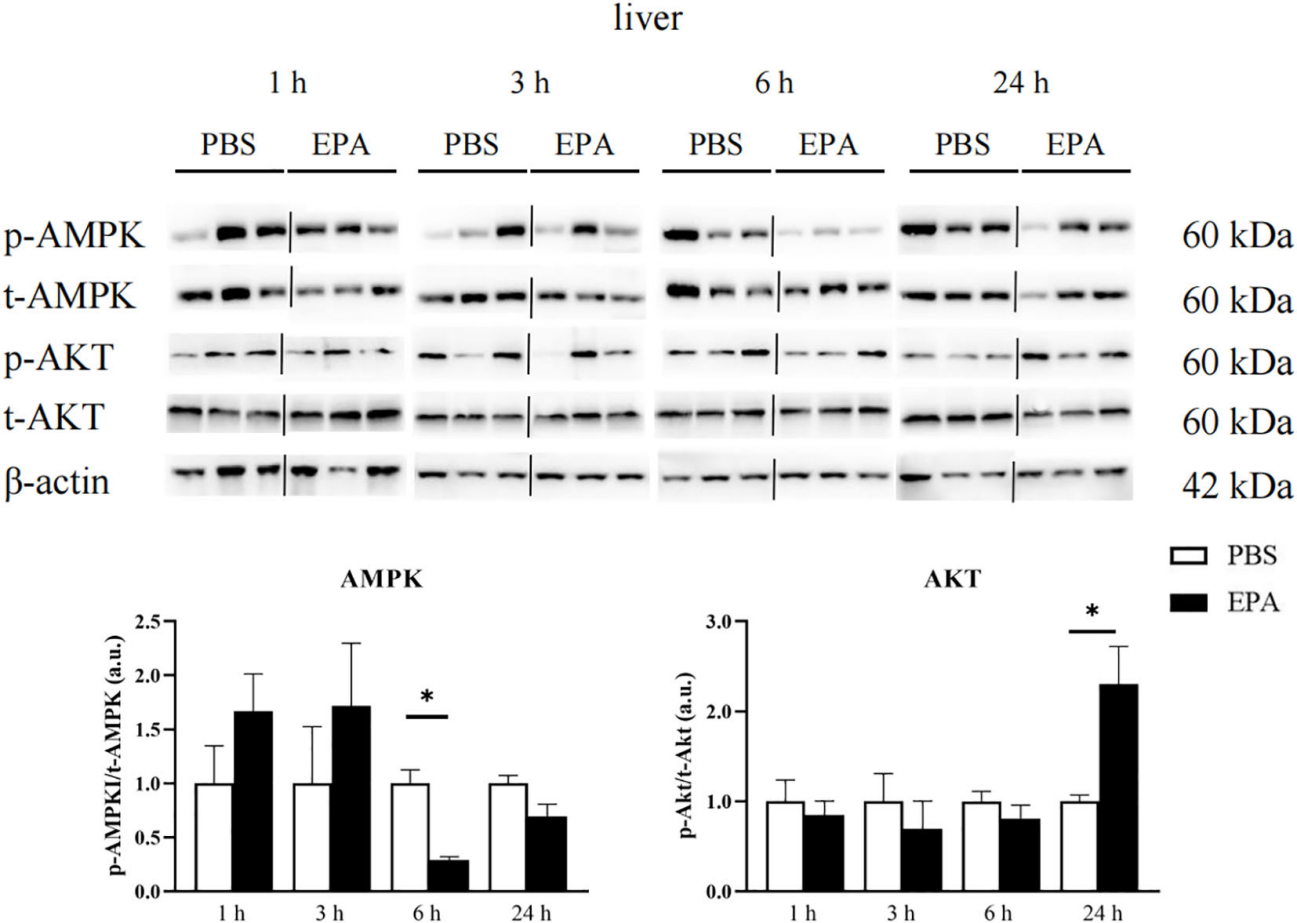
Effects of intraperitoneal administration of EPA on AMPK and AKT signaling pathways in tilapia liver. p-AMPK, phosphorylated AMPK protein; t-AMPK, total AMPK protein; p-AKT, phosphorylated AKT protein; t-AKT, total AKT protein; β-actin, β-actin protein. 1 h, 3 h, 6 h, and 24 h represent the tilapia groups that were sampled at 1 hour, 3 hours, 6 hours, and 24 hours, respectively. Mean ± standard error, n=3. * means significant differences, *p<0.05.

As AMPK and AKT signaling pathways play a very important role in food regulation, we investigated whether intraperitoneal injection of EPA affects AMPK and AKT signaling pathways in the hypothalamus and liver of tilapia after 24 h of fasting. Western blot results showed that p-AMPK/t-AMPK and p-AKT/t-AKT in the hypothalamus were significantly decreased at 6 h after intraperitoneal injection, indicating that AMPK and AKT signaling pathways were inhibited (**Figure 9**). The p-AMPK/t-AMPK in the liver decreased significantly at 6 h after intraperitoneal injection, indicating that the AMPK signaling pathway was inhibited. The p-AKT/t-AKT in the liver was significantly increased after intraperitoneal injection for 24 h, indicating that the AKT signaling pathway was activated (**Figure 10**).

## 4 Discussion

### 4.1 Short-term fasting down-regulated POMC protein signal in nLT of tilapia brain

The findings of this study demonstrated that a 24-hour fasting period leads to a reduction in POMC signaling within the nLT region and an increase in NPY signaling within the nPRG region. Our research team has previously elucidated the specific regional distribution of *pomc* and *npy* mRNA within the tilapia brain. The *pomc* expression is predominantly observed in the midbrain and diencephalon, with notable concentration in nLT, nPrG, and FR regions. Conversely, *npy* exhibits high expression levels within the telencephalon, as well as a presence in the preoptic area and midbrain, with particular concentration in nPPV, nLT, nPRG, among other regions (32). In other studies involving fish, *pomc* mRNA expression has typically been examined either across the entire brain or specifically within the hypothalamus. To date, no reports have been published regarding the detailed distribution pattern of POMC in specific brain regions of fish. Studies conducted on mammals have demonstrated that the expression of *pomc* mRNA is highly influenced by the body’s energy status, with fasting leading to a significant reduction in its expression (36). The nLT region of the fish brain is considered homologous to the mammalian hypothalamic arcuate nucleus, which plays a critical role in regulating feeding behavior (37). Following a 24-hour fasting period, there was a decrease in POMC signal within the nLT region of tilapia brain, suggesting that POMC in the nLT may contribute to the regulation of energy balance. However, further follow-up experiments are required to elucidate its specific mechanism. Studies on the distribution of NPY protein in brain regions, such as spotted gar (*Lepisosteus oculatus*) (38), cichlid fish (*Oreochromis mossambicus*) (39), catfish (*Clarias batrachus*) (40), and Senegalese sole (41) showed that NPY immunoreactive cells were found in the forebrain peripheral nerve, lateral nodular nucleus, periventricular organs and midbrain operculum, but NPY signals were not detected in these regions in this study. The variation in NPY signal distribution patterns observed across different studies may be attributed to the limited specificity of NPY antibodies. Due to its shared sequence with other pancreatic peptide families, such as PYY, NPY antibodies might exhibit cross-reactivity with peptides from the same family. In contrast to the findings of this study, Yokobori et al. demonstrated a significant increase in NPY signals within the nPPv and nLT regions after 7 days of fasting in zebrafish through immunohistochemistry (9).

### 4.2 Short-term fasting activates the fatty acid sensing system in the hypothalamus and liver of tilapia

So far, studies on the fatty acid sensing system in fish have been mainly carried out by feeding a high-fat diet, intraperitoneal and intracerebral injection of fatty acids, and *in vitro* hypothalamic fragment incubation of fatty acids (23, 24, 42). This study is the first to study the fatty acid sensing system of fish by short-term fasting. The findings revealed that following short-term fasting, the mechanisms responsible for fatty acid metabolism and mitochondrial activity perception in the hypothalamus were activated at 9 hours but inhibited after 24 hours. However, no significant changes were observed in the perception mechanisms of CD36, activated protein kinase C, and lipoprotein lipase. In the liver, the pathways involved in fatty acid metabolism and lipoprotein lipase sensing were inhibited, while those related to CD36, mitochondrial activity, and activated protein kinase C sensing mechanisms were activated. These results suggest that a fatty acid sensing system exists in both the hypothalamus and liver of tilapia; however, fasting affects different fatty acids in tilapia serum to varying degrees. Metabolomics analysis demonstrated a significant increase in levels of long-chain saturated and monounsaturated fatty acids after short-term fasting, while long-chain polyunsaturated fatty acids decreased significantly. Therefore, it is difficult to determine which specific fatty acids can activate or inhibit the fatty acid sensing system in both the hypothalamus and liver of tilapia. Studies conducted on mammals have demonstrated that long-chain fatty acids possess the ability to activate the fatty acid sensing system, whereas medium-chain fatty acids lack this capability. In rainbow trout, treatment with long-chain fatty acid OA has been observed to stimulate the activation of the fatty acid sensing system in the hypothalamus, brucella, and liver (23, 43). Additionally, the researchers discovered that in rainbow trout, treatment with medium-chain fatty acids can elicit reactions similar to those induced by OA treatment, contradicting previous findings observed in mammals (44). The activation of the fish fatty acid sensing system by medium-chain fatty acids may be attributed to the high abundance of such fatty acids in fish fat, as well as the absence of a clear oxidative preference for long-chain and medium-chain fatty acids in fish (45, 46). In addition to medium-chain and long-chain fatty acids, α-linolenic acid, a type of long-chain polyunsaturated fatty acid, can also activate the hypothalamic fatty acid sensing system in Senegalese sole. However, another long-chain fatty acid EPA fails to elicit the same response (25). The distinction lies in the fact that intraperitoneal injection of EPA in European sea bass triggers activation of the hypothalamic fatty acid sensing system (31). It is evident that the response to specific long-chain unsaturated fatty acids varies among different fish species.

### 4.3 Tilapia may increase EPA catabolism during short-term fasting

The findings of this investigation indicate that long-chain fatty acids are the most prevalent differential metabolites in tilapia serum following a 24-hour fast. Previous research has demonstrated that the hierarchy of fatty acid oxidation in most fish is saturated > monounsaturated > polyunsaturated > long-chain polyunsaturated (31). After a period of 45 days of fasting, there was a significant decrease in the levels of monounsaturated fatty acids in the liver and muscle tissues of hybrid red tilapia (*Oreochromis mossambicus* X O. *niloticus*), while there was a notable increase in the levels of polyunsaturated fatty acids (47). However, variations in species, dietary composition, and stress treatments such as fasting can alter the hierarchy of fatty acid oxidation. Bou et al. discovered a significant increase in the level of monounsaturated fatty acid 18:1n-9 in Atlantic salmon adipocytes following fasting compared to saturated fatty acid 16:0 and polyunsaturated fatty acid EPA (48). After 27 days of fasting, the whole-body EPA of Chinook salmon decreased significantly (29); EPA in the liver of Atlantic salmon decreased significantly after 86 days of fasting (49). Arachidonic acid belongs to ω-6 polyunsaturated fatty acids, which play an important role in maintaining the structure and function of cell membrane. EPA is classified as an ω-3 polyunsaturated fatty acid and exhibits a strong physiological association with fish, contributing to the regulation of biofilm structure and immune system function. The results from serum metabolomics analysis revealed a significant increase in levels of most long-chain saturated and monounsaturated fatty acids among differential metabolites after 24 hours of fasting. Conversely, there was a notable decrease in levels of arachidonic acid and EPA. This observation suggests that lipolysis occurring in peripheral tissues rich in lipid content (such as fat and liver) releases more fatty acids into circulation than those taken up by other peripheral tissues for oxidative energy supply, leading to elevated levels of long-chain saturated and monounsaturated fatty acids. The reduction in arachidonic acid and EPA levels may be attributed to their higher uptake rate by other peripheral tissues from blood circulation, in comparison to their lipid breakdown rate. Additionally, during fastingn periods, animals tend to preferentially utilize ω-3 long-chain polyunsaturated fatty acids rather than saturated or monounsaturated fatty acids to meet energy requirements (15). Therefore, it can be speculated that EPA, being an ω-3 long-chain polyunsaturated fatty acid, plays a vital physiological role during short-term fasting in tilapia.

### 4.4 EPA activated tilapia fatty acid sensing system

The findings of this study demonstrated a significant reduction in food intake in tilapia after 24 hours following intraperitoneal administration of EPA. Consistent with the observed changes in food intake, there was a significant increase in *pomc* mRNA levels within the hypothalamus of tilapia at 3, 6, and 24 hours post-intraperitoneal injection of EPA. In contrast to the effects observed in tilapia, intraperitoneal administration of EPA did not impact food intake or mRNA levels of *npy* and *pomc* within the hypothalamus of Senegal sole and European seabass (25, 31). We hypothesize that the impact of EPA on fish food intake may exhibit species-specific variations. Previous research has demonstrated that excessive lipid consumption can influence the feeding regulation in fish. High-fat diet studies conducted on various fish species, such as rainbow trout (50), chinook salmon (51) and Senegalese sole (52), have consistently shown a significant reduction in food intake. Additionally, intraperitoneal or intracerebral administration of nOA or OA in rainbow trout has been found to significantly decrease their food intake (23, 53). Similar to findings in mammals, studies in fish have indicated that activation of the fatty acid sensing system can modulate food intake by altering the expression of feeding-related neuropeptides (54). In rainbow trout, high-fat diet feeding led to a significant increase in *pomc* mRNA levels within the hypothalamus (28), while grass carp exhibited a notable decrease in *npy* mRNA levels within their hypothalamus following high-fat diet consumption (27). However, no significant changes were observed regarding *npy* mRNA levels within the hypothalamus of rainbow trout (50). In addition, intraperitoneal injection and intracerebral injection of OA in rainbow trout caused a decrease in the level of *npy* mRNA and an increase in the level of *pomc* mRNA in the hypothalamus (23, 24). Total cholesterol (T-CHO), triglycerides (TG), low-density lipoprotein cholesterol (LDL-C), high-density lipoprotein cholesterol (HDL-C), and glucose are closely related to the glucose and lipid metabolism in fish (55, 56). After intraperitoneal administration of EPA to tilapia following short-term fasting, there was a significant increase in serum T-CHO levels after 6 hours, while the levels of glucose, TG, LDL-C, and HDL-C did not show significant changes. Consistent with our findings, a study on European seabass demonstrated that EPA significantly elevated serum T-CHO levels 3 hours post intraperitoneal injection but had no impact on serum glucose, TG, or phospholipid levels (31). In Senegalese sole, it was observed that EPA led to a significant increase in serum TG levels 3 hours after intraperitoneal injection (25). These differences may be attributed to variations in fish species’ sensitivity and response towards fatty acids. Furthermore, we also noted that EPA significantly reduced serum very-low-density lipoprotein cholesterol (VLDL-C) levels at both 6 and 24 hours post intraperitoneal injection. To date, no studies have investigated the effect of intraperitoneally administered fatty acids on fish’s serum VLDL-C levels. Since VLDL-C is responsible for transporting TG out of the liver, we speculate that EPA acts as a satiety signal when injected into fish’s peritoneum by indicating an energy surplus state and inhibiting the transport of TG from the liver to other tissues for energy supply; thus resulting in a notable decrease in serum VLDL-C levels.

After intraperitoneal injection of EPA in tilapia, the mRNA level of *fas*, a gene related to fatty acid sensing system regulated by fatty acid metabolism, was significantly down-regulated in hypothalamus. The mRNA level of *lxrα*, a fatty acid sensing system-related gene regulated by CD36, was significantly down-regulated, and the mRNA level of *pparα* did not change significantly. The mRNA level of *cpt1α*, a gene related to mitochondrial activity-related fatty acid sensing system, was significantly down-regulated, but the expression level of *hadh* did not change significantly. PKC-regulated fatty acid sensing system-related gene *kir10* and lipoprotein lipase activity-regulated fatty acid sensing system-related gene *lpl* mRNA levels did not change significantly. The results obtained from European seabass indicated that intraperitoneal administration of EPA had no impact on the mRNA levels of *fas*, *lxrα*, and *srebp1* in the hypothalamus. However, it significantly enhanced the activity of malic enzyme and HOAD, suggesting a potential involvement of EPA in the regulation of fatty acid metabolism (31). The researchers have discovered that the monounsaturated fatty acid OA and the polyunsaturated fatty acid linolenic acid are capable of activating the hypothalamus’ fatty acid sensing system, whereas the polyunsaturated fatty acid EPA lacks this ability (25). The carbon chain of EPA is longer compared to Linolenic acid, both of which are classified as ω-3 PUFA. While Linolenic acid can activate the hypothalamic fatty acid sensing system in Senegalese sole as an ω-3 PUFA, further exploration is needed to understand why EPA cannot (25). At present, the exploration of the fatty acid sensing system in fish liver is limited to rainbow trout (53). This study has found that in tilapia, after intraperitoneal injection of EPA, the mRNA level of the fatty acid sensing system gene *fas* related to fatty acid metabolism regulation in the liver significantly decreased. In contrast, in rainbow trout, the mRNA level of *fas* did not show significant changes after intraperitoneal injection of long-chain fatty acid OA (oleic acid) and medium-chain fatty acid nOA (n-Octanoic acid) (53). Additionally, intraperitoneal injection of EPA in tilapia led to a significant decrease in the mRNA level of the fatty acid sensing system-related gene *srebpc1*, but had no significant effect on the mRNA levels of *lxrα* and *pparα*. In rainbow trout, however, intraperitoneal injection of OA and nOA did not result in significant changes in the mRNA levels of *srebpc1*, *lxrα*, and *pparα* (53). Given the extremely limited research on the fatty acid sensing system in fish liver, we are unable to clearly explain the reasons for the differences in the effects of intraperitoneal injection of fatty acids on the fatty acid sensing system in the liver of tilapia and rainbow trout. It is speculated that this difference may be caused by the different types of fatty acids and the differences between fish species. In mammals, this system is specifically activated by long-chain fatty acids and remains unresponsive to medium-chain or short-chain fatty acids (17). However, in fish, a distinction arises as both medium-chain fatty acid nOA and long-chain fatty acid OA have been observed to activate the fatty acid sensing system in rainbow trout (53). The researchers hypothesize that this may be attributed to the high concentration of medium-chain fatty acids in the adipose tissue of teleost fish (46). Therefore, the current research on fatty acid sensing systems in fish peripheral tissues solely focuses on elucidating the commonalities and disparities between long-chain fatty acid OA and medium-chain fatty acid nOA in their activation of such systems (53, 57). The present study represents the pioneering attempt to investigate the impact of EPA on the peripheral tissue’s fatty acid sensing system in fish, revealing that administration of EPA resulted in a certain level of activation within the liver’s fatty acid sensing system.

### 4.5 The relationship between fatty acid perception and AMPK and AKT in tilapia

Studies in mammals have shown that AMPK plays a role in lipid metabolism and energy homeostasis. Activation of AMPK suppresses SREBP, a crucial transcription factor involved in fatty acid biosynthesis, while simultaneously stimulating fatty acid oxidation (58). Moreover, it inhibits acetyl-CoA carboxylase activity, thereby alleviating the inhibition of CPT1 and ultimately leading to enhanced fatty acid oxidation (59). In fish, studies have demonstrated the involvement of AMPK and AKT in the regulation of lipid metabolism (60, 61). However, intraperitoneal injection of EPA in this study did not elicit any effects on the signaling pathways of AMPK and AKT in the hypothalamus of tilapia. Similarly, feeding rainbow trout with a high-fat diet did not alter the phosphorylation level of AMPK in their hypothalamus (28). Conversely, incubation of rainbow trout’s hypothalamus with OA and nOA significantly enhanced AKT phosphorylation while inhibiting AMPK phosphorylation (62). The findings from our study revealed that after 6 hours of intraperitoneal EPA injection, there was a significant decrease in p-AMPK/t-AMPK ratio and a significant increase in p-AKT/t-AKT ratio observed in tilapia liver. After treatment with OA or nOA, rainbow trout primary hepatocytes exhibited significant activation of the AKT signaling pathway and inhibition of the AMPK signaling pathway (63). Additionally, high-fat diet feeding resulted in a notable increase in liver phosphorylation levels of AKT, thereby activating the AKT signaling pathway. The researchers postulated that this activation would enhance hepatic lipid production rates. We hypothesized that EPA may serve as a satiety signal for tilapia; thus, intraperitoneal injection of EPA leads to nutrient compensation and perception of the satiety signal by fish. Consequently, this inhibits AMPK phosphorylation in the liver while activating the AKT signaling pathway, ultimately promoting an increased rate of liver lipid production and facilitating excessive fatty acid consumption.

The current study demonstrated that the administration of EPA affected the AMPK and AKT signaling pathways in the liver of tilapia, but did not influence these pathways in the hypothalamus. This distinction may be attributed to the tissue-specific roles and distinct mechanisms by which these signaling pathways respond to nutrients. As a primary metabolic organ, the liver plays a pivotal role in regulating the metabolism of lipids, proteins, and carbohydrates (64). Activation of AMPK in this organ promotes fatty acid oxidation, inhibits cholesterol synthesis, and reduces lipogenesis, thereby enhancing lipid metabolism (65). Additionally, AKT is intricately involved in the regulation of glucose uptake and gluconeogenesis, processes essential for maintaining blood glucose homeostasis (66). Conversely, while the hypothalamus is crucial for the regulation of appetite, body weight, and endocrine functions, its primary role does not directly involve metabolic regulation. In this region, the AMPK and AKT signaling pathways are more engaged in modulating energy balance and food intake responses rather than executing direct metabolic control (67, 68). To further elucidate the mechanisms underlying EPA’s selective effects on specific signaling pathways within distinct tissues, it is imperative to conduct more comprehensive investigations focusing on tissue-specifically expressed receptors, enzymes, and other molecular mechanisms.

## 5 Conclusions

In summary, this study investigated the impact of a 24-hour fasting period on NPY and POMC in the tilapia brain and identified POMC in nLT as a potential key player in tilapia feeding behavior and energy regulation. The results from serum metabolomics revealed that fasting significantly increased the levels of most long-chain fatty acids, while long-chain polyunsaturated fatty acid EPA was notably reduced, suggesting its involvement in tilapia’s perception system for fatty acids. Transcriptional analysis of both hypothalamus and liver demonstrated that fasting activated the fatty acid sensing system in these organs. Furthermore, EPA compensation effectively inhibited the AMPK signaling pathway in both hypothalamus and liver, thereby regulating food intake through modulation of *pomc* mRNA expression level in the hypothalamus. These findings highlight EPA as an important satiation factor for tilapia.

## Data Availability

The original contributions presented in the study are included in the article/**Supplementary Material**, further inquiries can be directed to the corresponding author/s.
