# Supplementary material for: Fatty acid metabolism after short-term fasting: POMC response and EPA signal maintain homeostasis in tilapia
Source: Front Endocrinol (Lausanne). 2025 May 9;16:1585216. doi: 10.3389/fendo.2025.1585216 (PMC12098032; doi:10.3389/fendo.2025.1585216)
Supplement: Supplementary file 1 [file DataSheet1.zip › Image explanation.docx]

Image explanation

On all the original western blot images provided, there are three treatment groups. The PBS treatment group and the EPA treatment group are the ones we intend to feature in the manuscript. The results of the other treatment group are not relevant to the manuscript we are providing. Consequently, when submitting the results file, we omitted the results of the irrelevant treatment group and included only those of the PBS and EPA treatment groups.

The “p-AKT_1h_hypothalamus” image is used as an example for illustration.

Original picture “p-AKT_1h_hypothalamus” is shown below.


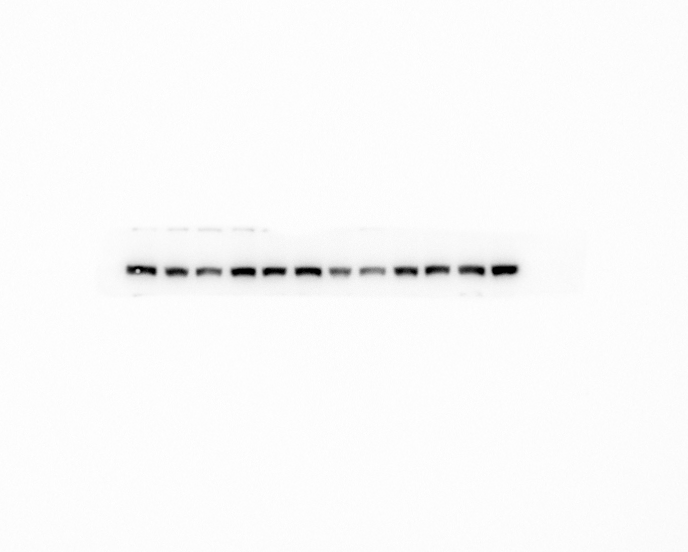


EPA

Other treatment

PBS

The processed images (only the PBS and EPA-treated groups retained) are shown below.

EPA

PBS


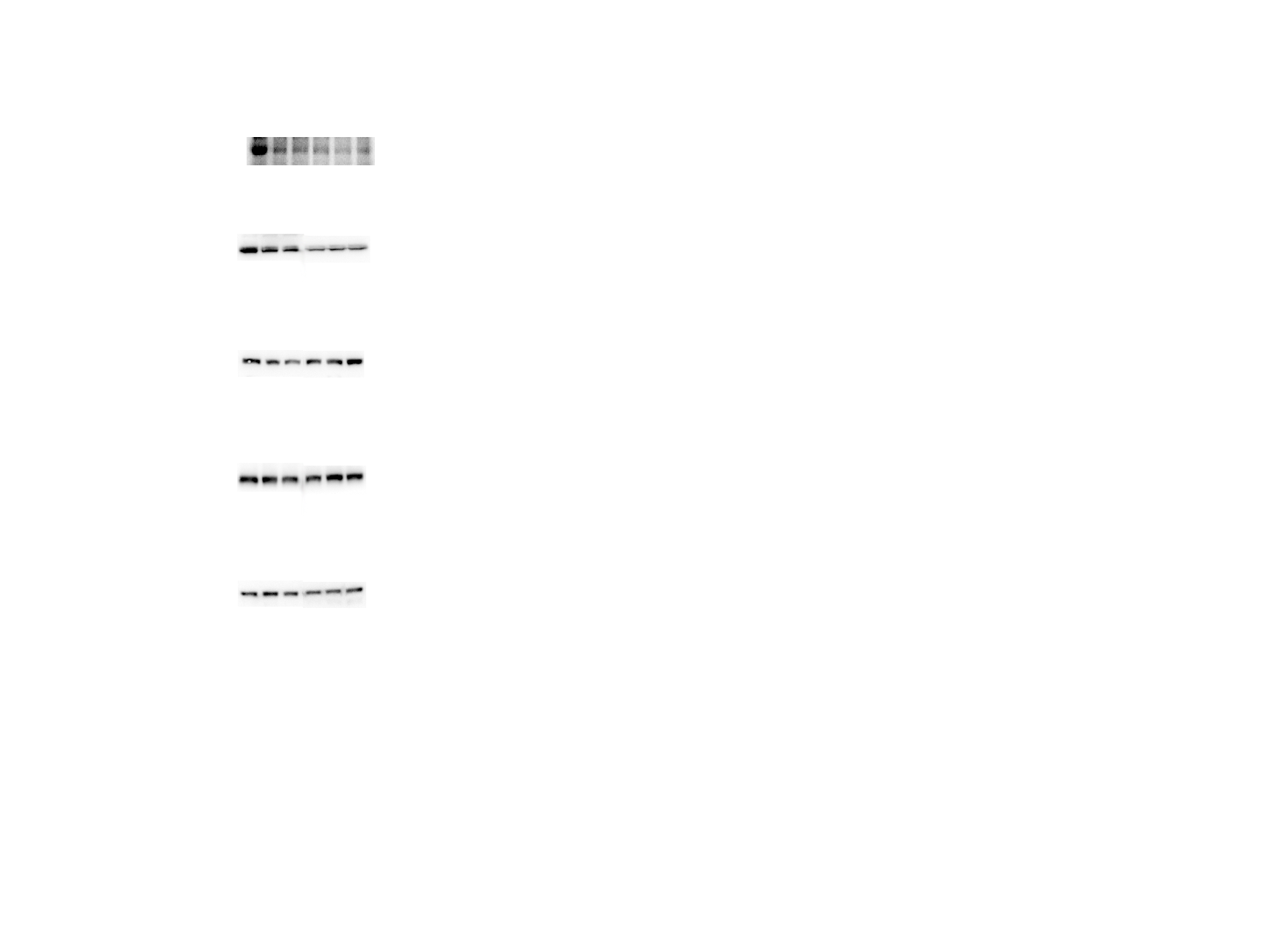


With regard to the microscopy images, we have supplied both the full image and the local enlarged image, with the precise location of the local enlarged image indicated within the full image. Consequently, we have no further material to provide as a supplement.
