# Supplementary figures and images for: Fatty acid metabolism after short-term fasting: POMC response and EPA signal maintain homeostasis in tilapia
Source: Front Endocrinol (Lausanne). 2025 May 9;16:1585216. doi: 10.3389/fendo.2025.1585216 (PMC12098032; doi:10.3389/fendo.2025.1585216)

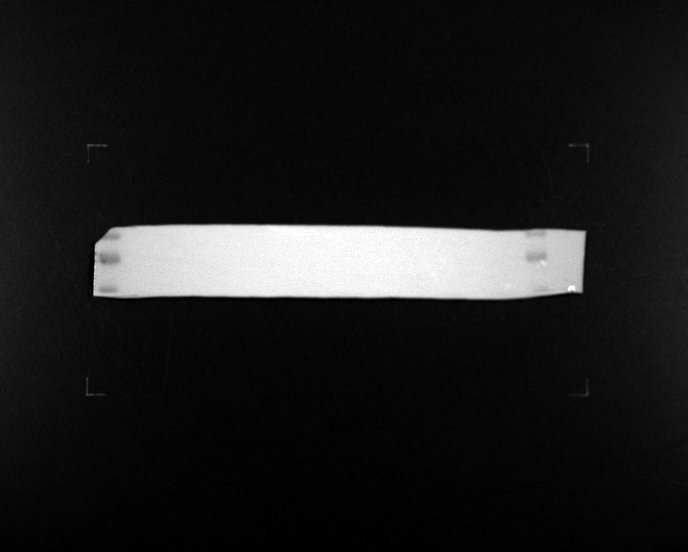

Supplement: Supplementary file 1 [file DataSheet1.zip › AKT_Hypothalamus/p-AKT_1h_hypothalamus bright field.tif]

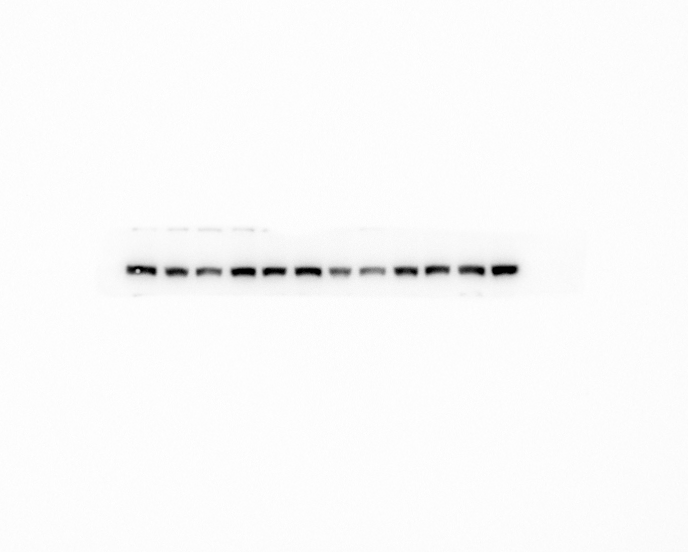

Supplement: Supplementary file 1 [file DataSheet1.zip › AKT_Hypothalamus/p-AKT_1h_hypothalamus.tif]

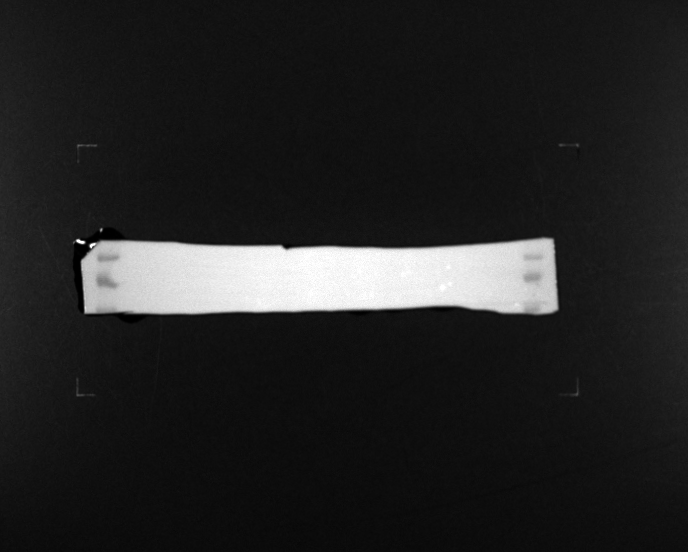

Supplement: Supplementary file 1 [file DataSheet1.zip › AKT_Hypothalamus/p-AKT_24h_hypothalamus bright field.tif]

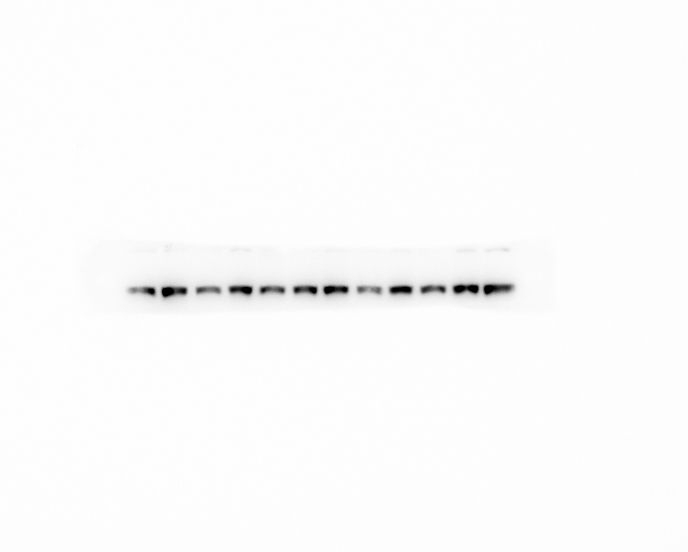

Supplement: Supplementary file 1 [file DataSheet1.zip › AKT_Hypothalamus/p-AKT_24h_hypothalamus.tif]

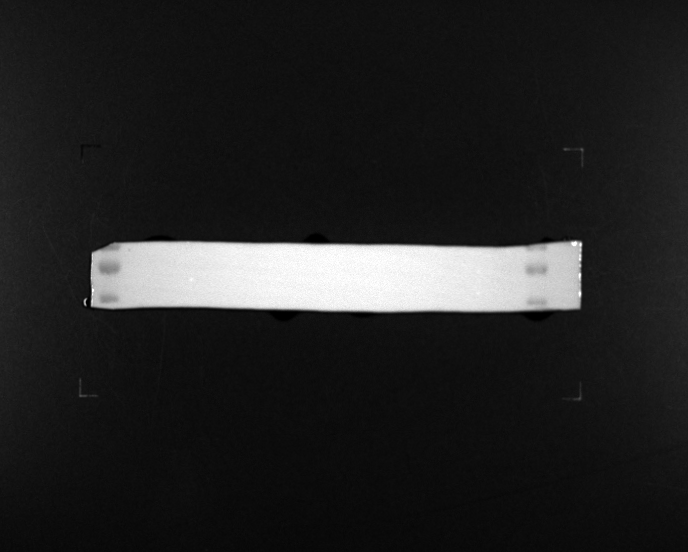

Supplement: Supplementary file 1 [file DataSheet1.zip › AKT_Hypothalamus/p-AKT_3h_hypothalamus bright field.tif]

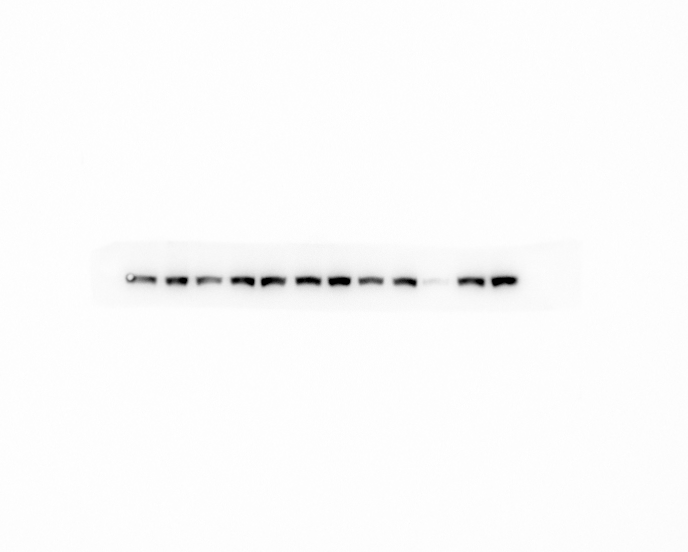

Supplement: Supplementary file 1 [file DataSheet1.zip › AKT_Hypothalamus/p-AKT_3h_hypothalamus.tif]

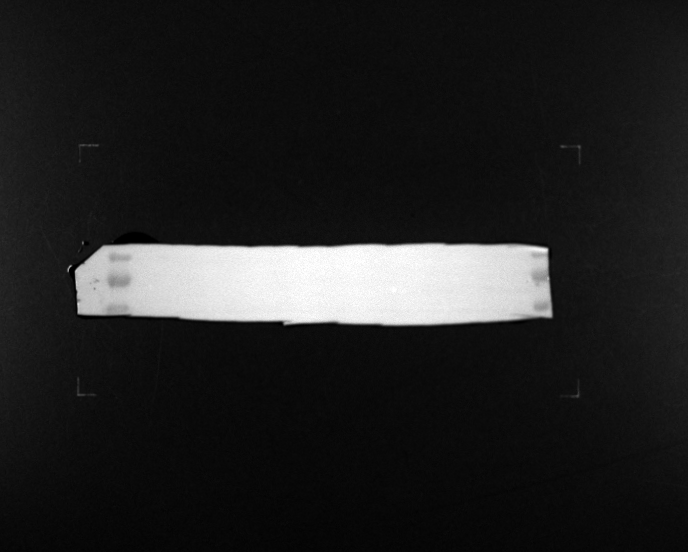

Supplement: Supplementary file 1 [file DataSheet1.zip › AKT_Hypothalamus/p-AKT_6h_hypothalamus bright field.tif]

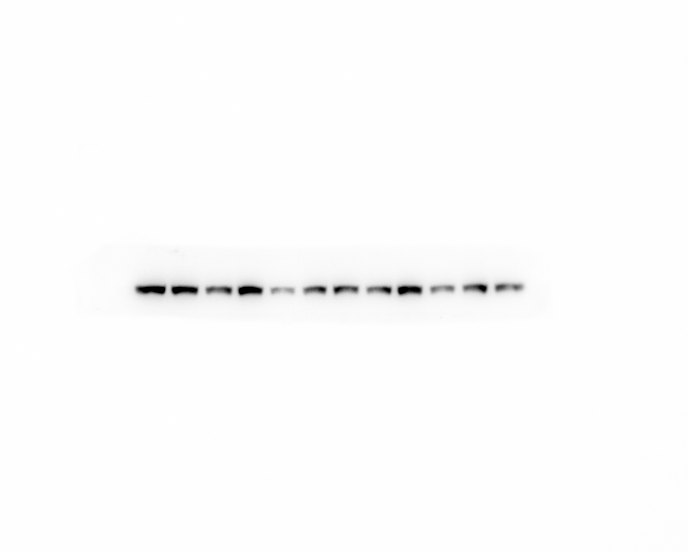

Supplement: Supplementary file 1 [file DataSheet1.zip › AKT_Hypothalamus/p-AKT_6h_hypothalamus.tif]

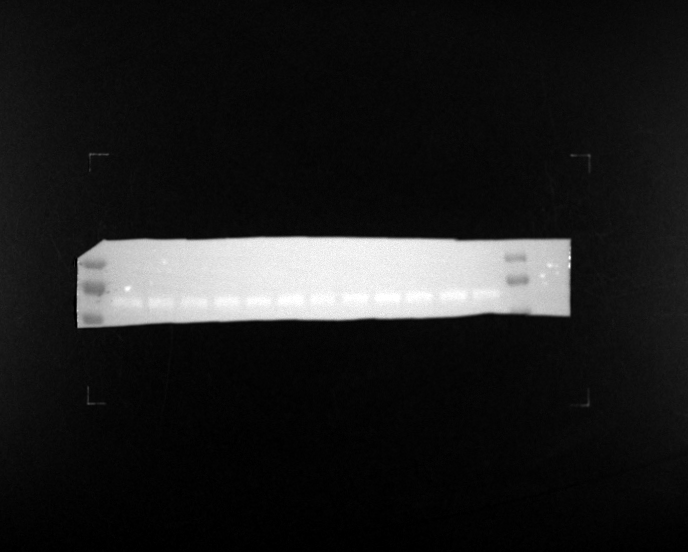

Supplement: Supplementary file 1 [file DataSheet1.zip › AKT_Hypothalamus/t-AKT_1h_hypothalamus bright field.tif]

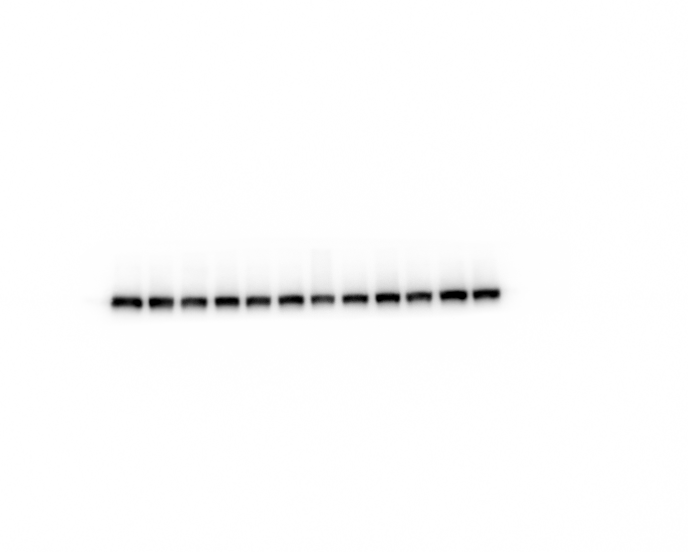

Supplement: Supplementary file 1 [file DataSheet1.zip › AKT_Hypothalamus/t-AKT_1h_hypothalamus.tif]

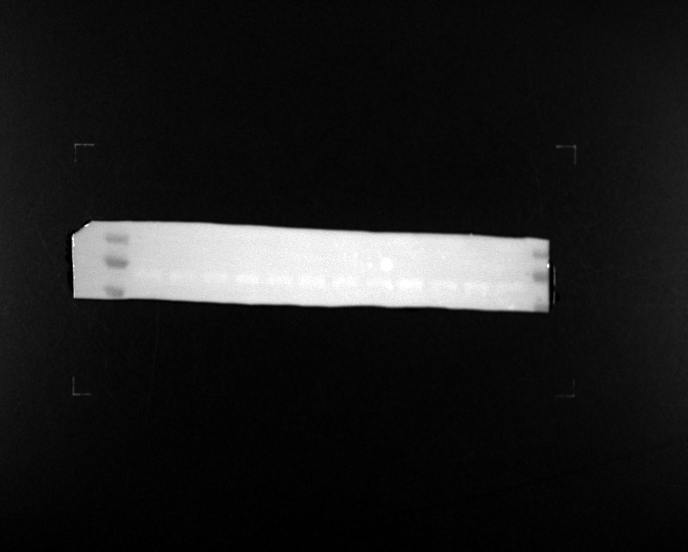

Supplement: Supplementary file 1 [file DataSheet1.zip › AKT_Hypothalamus/t-AKT_24h_hypothalamus bright field.tif]

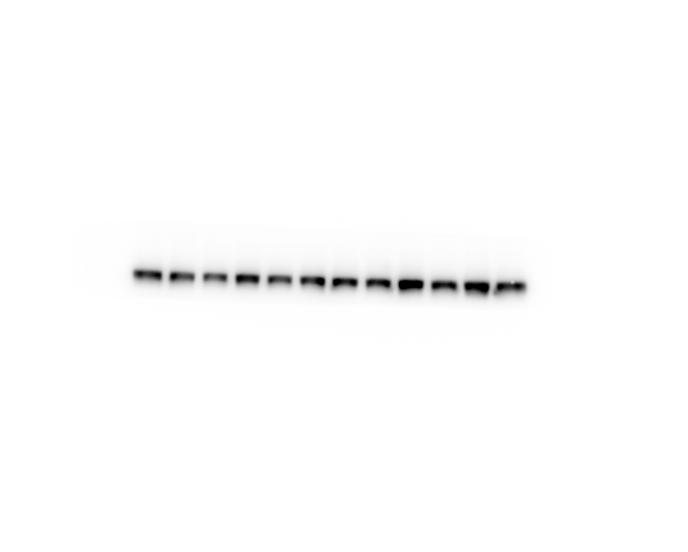

Supplement: Supplementary file 1 [file DataSheet1.zip › AKT_Hypothalamus/t-AKT_24h_hypothalamus.tif]

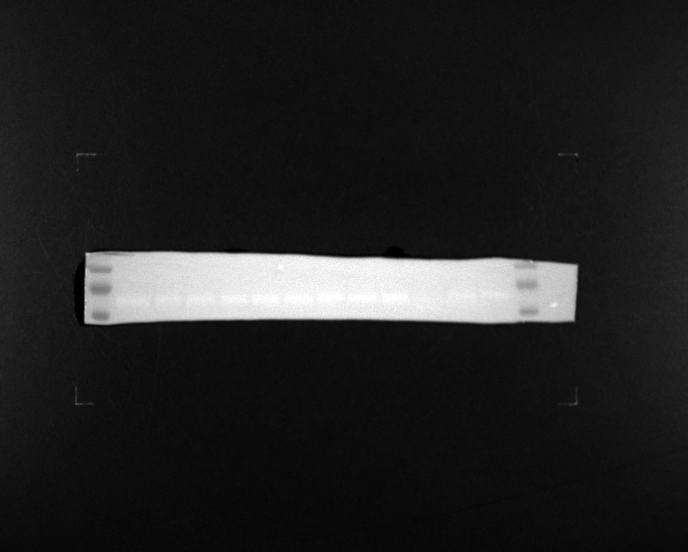

Supplement: Supplementary file 1 [file DataSheet1.zip › AKT_Hypothalamus/t-AKT_3h_hypothalamus bright field.tif]

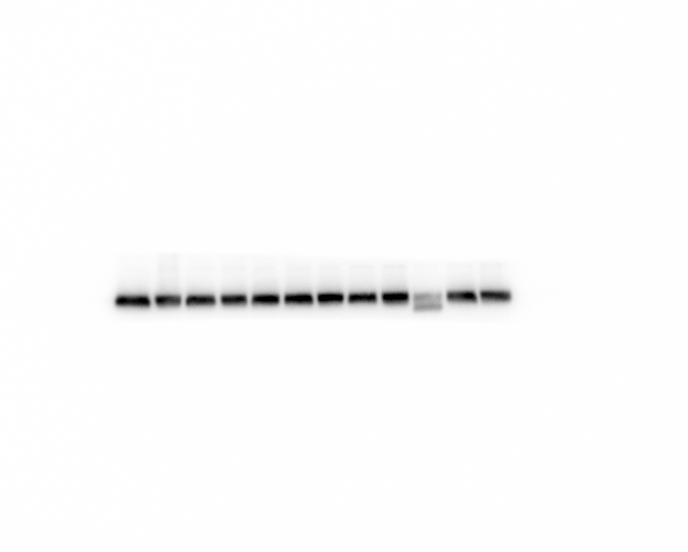

Supplement: Supplementary file 1 [file DataSheet1.zip › AKT_Hypothalamus/t-AKT_3h_hypothalamus.tif]

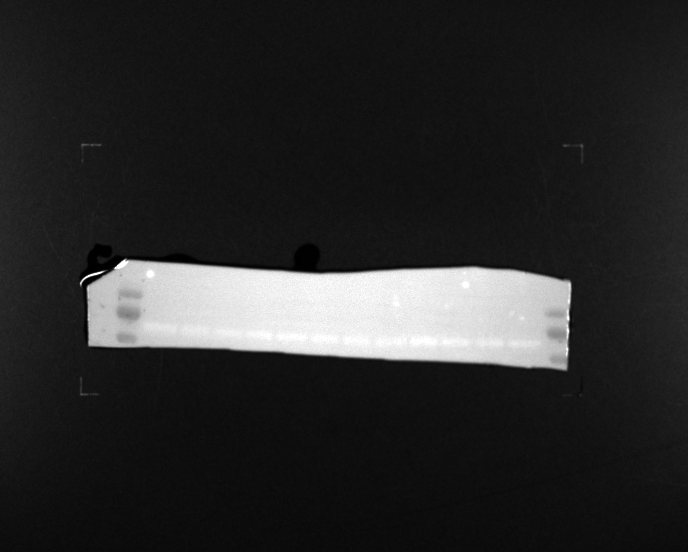

Supplement: Supplementary file 1 [file DataSheet1.zip › AKT_Hypothalamus/t-AKT_6h_hypothalamus bright field.tif]

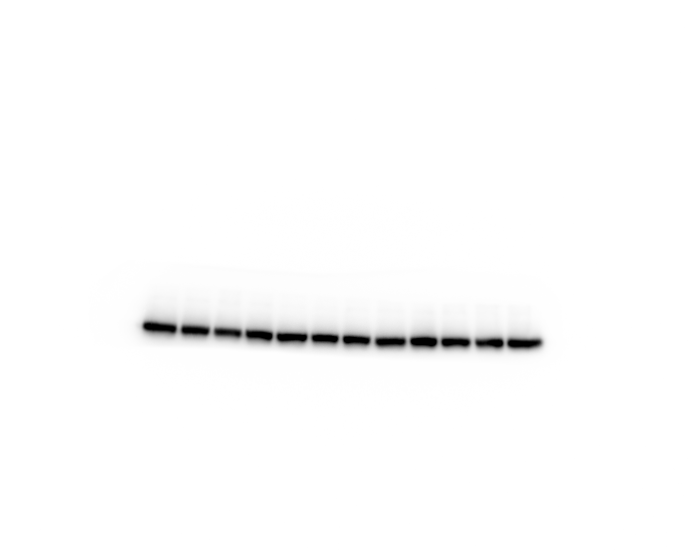

Supplement: Supplementary file 1 [file DataSheet1.zip › AKT_Hypothalamus/t-AKT_6h_hypothalamus.tif]

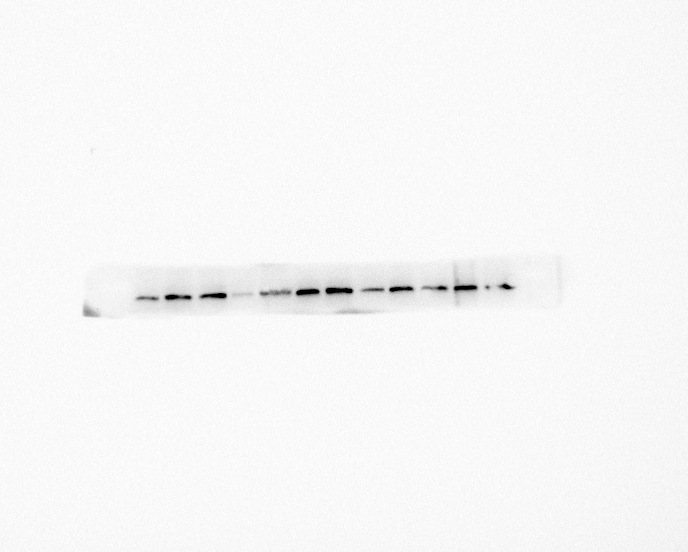

Supplement: Supplementary file 1 [file DataSheet1.zip › AKT_LIVER/p-AKT_1h_liver.tif]

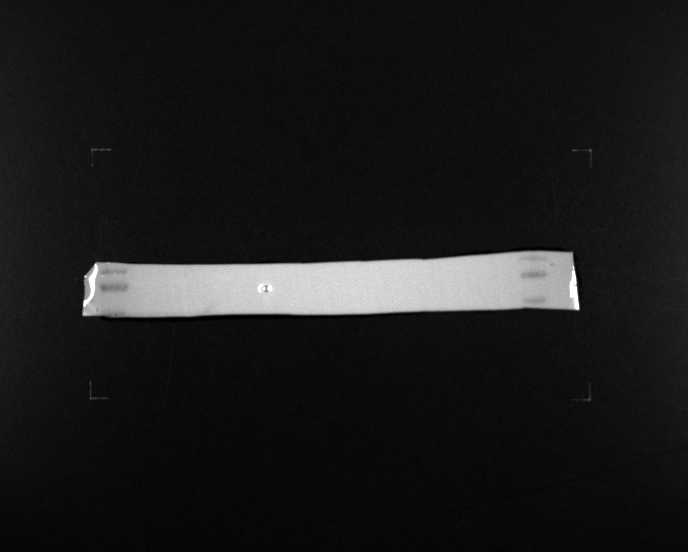

Supplement: Supplementary file 1 [file DataSheet1.zip › AKT_LIVER/p-AKT_1h_liver_bright field.tif]

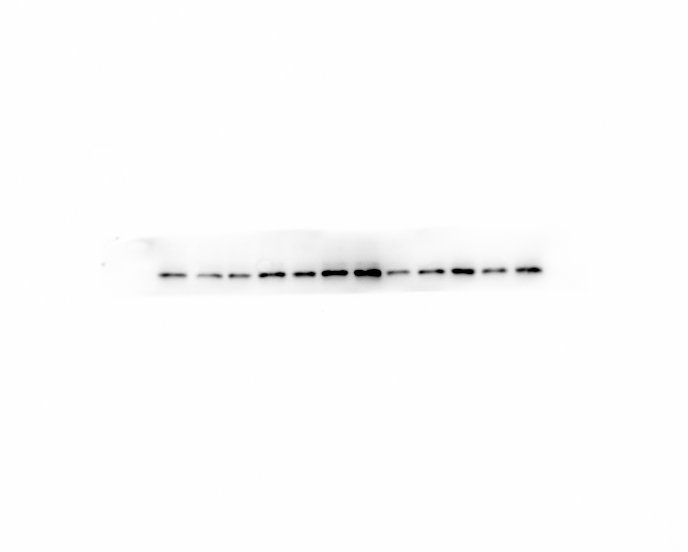

Supplement: Supplementary file 1 [file DataSheet1.zip › AKT_LIVER/p-AKT_24h_liver.tif]

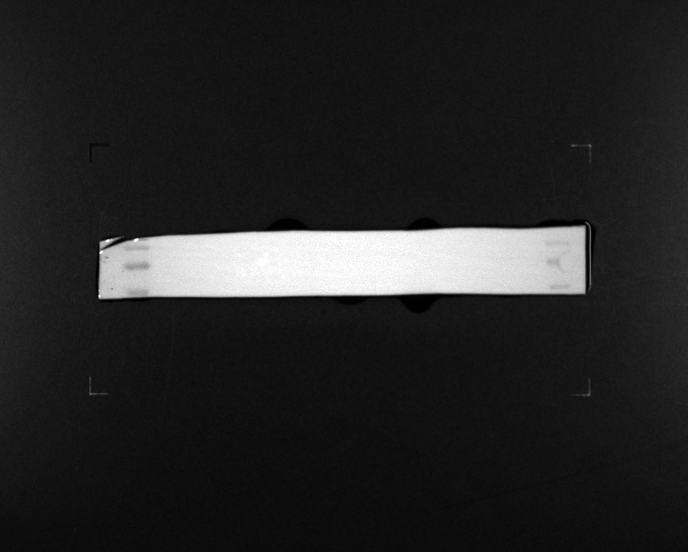

Supplement: Supplementary file 1 [file DataSheet1.zip › AKT_LIVER/p-AKT_24h_liver_bright field.tif]

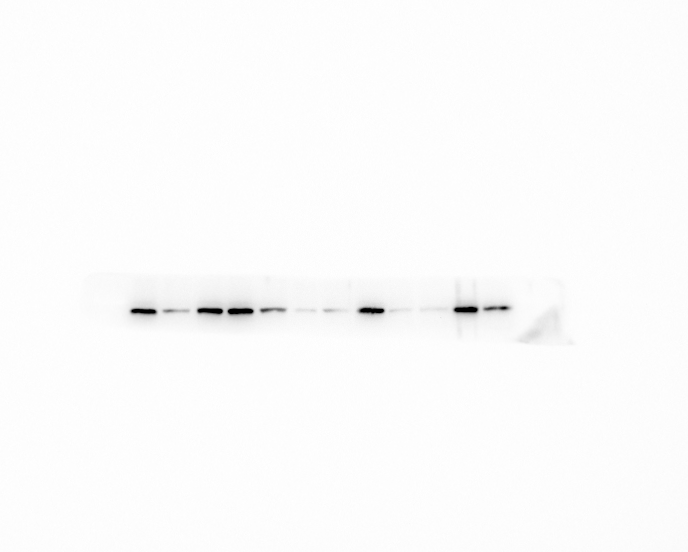

Supplement: Supplementary file 1 [file DataSheet1.zip › AKT_LIVER/p-AKT_3h_liver.tif]

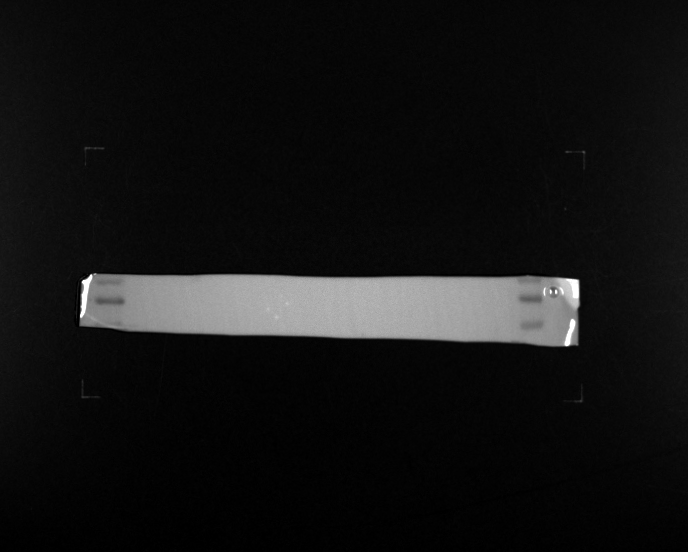

Supplement: Supplementary file 1 [file DataSheet1.zip › AKT_LIVER/p-AKT_3h_liver_bright field.tif]

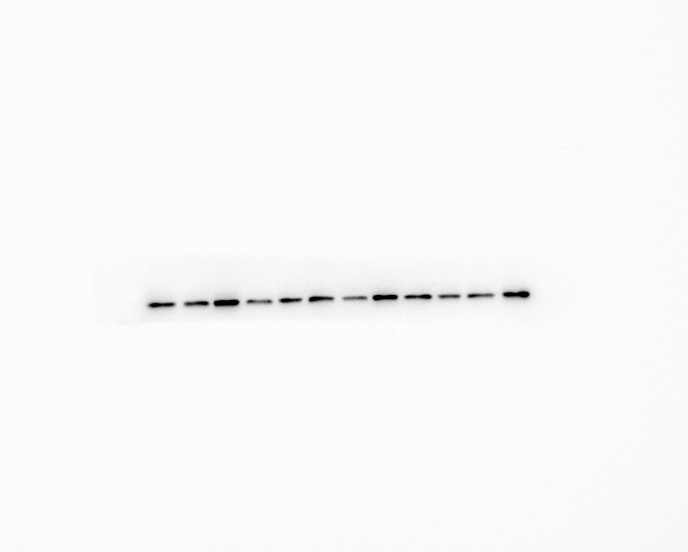

Supplement: Supplementary file 1 [file DataSheet1.zip › AKT_LIVER/p-AKT_6h_liver.tif]

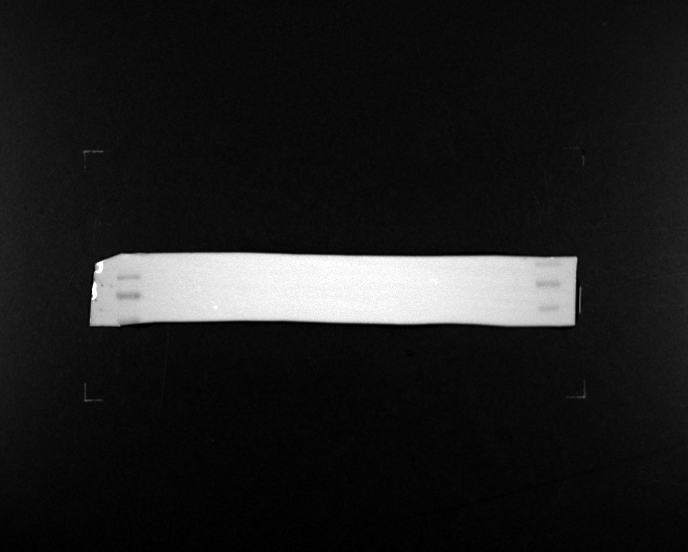

Supplement: Supplementary file 1 [file DataSheet1.zip › AKT_LIVER/p-AKT_6h_liver_bright field.tif]

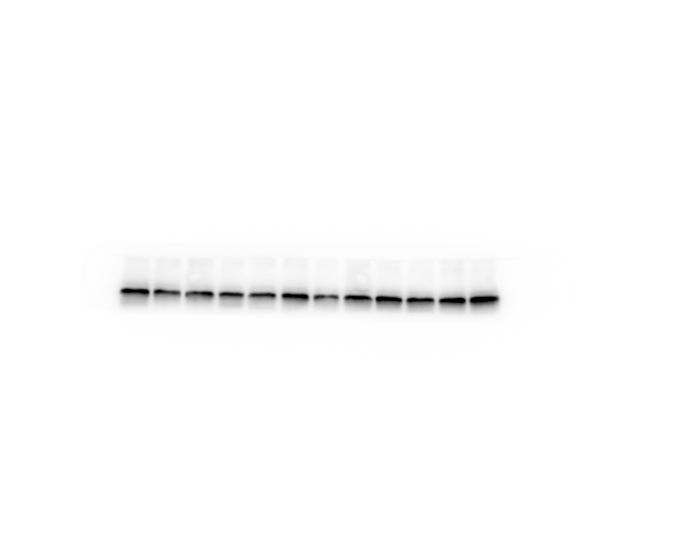

Supplement: Supplementary file 1 [file DataSheet1.zip › AKT_LIVER/t-AKT_1h_liver.tif]

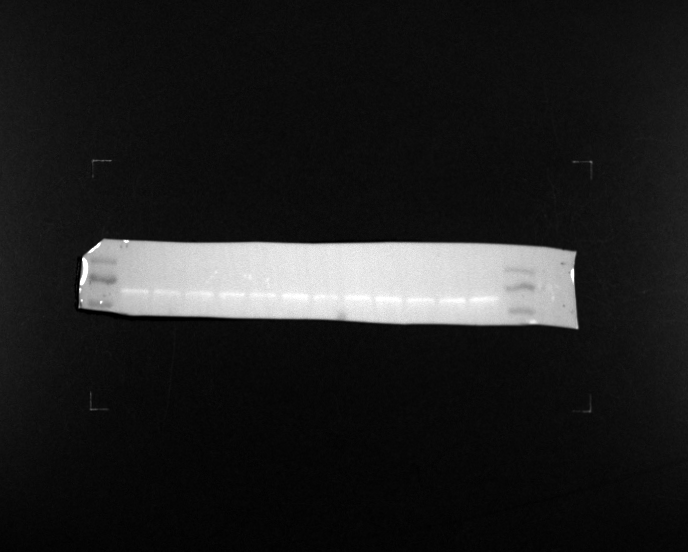

Supplement: Supplementary file 1 [file DataSheet1.zip › AKT_LIVER/t-AKT_1h_liver_bright field.tif]

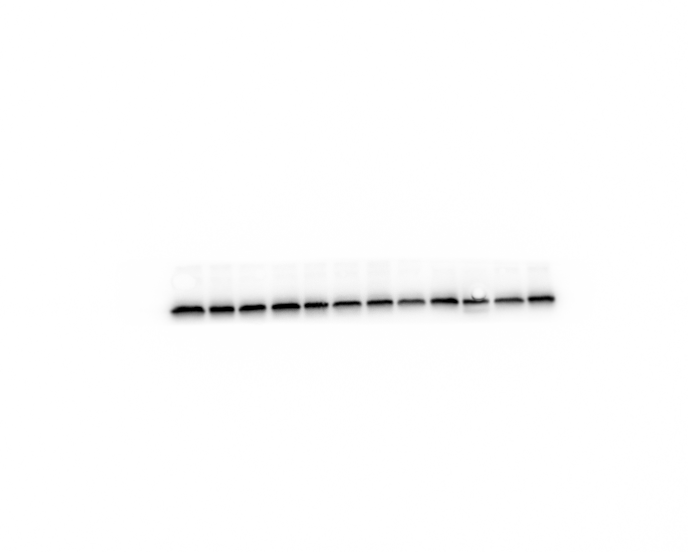

Supplement: Supplementary file 1 [file DataSheet1.zip › AKT_LIVER/t-AKT_24h_liver.tif]

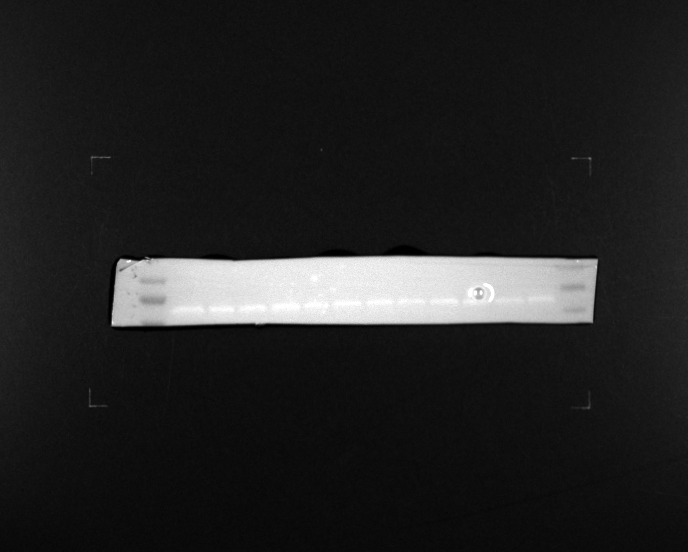

Supplement: Supplementary file 1 [file DataSheet1.zip › AKT_LIVER/t-AKT_24h_liver_bright field.tif]

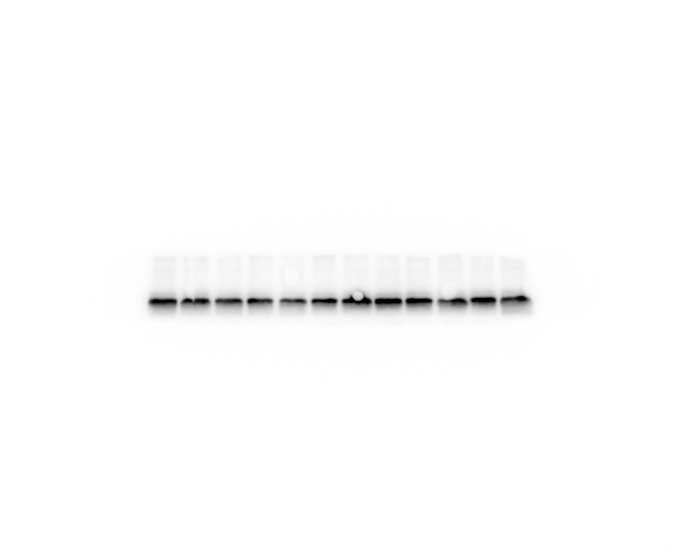

Supplement: Supplementary file 1 [file DataSheet1.zip › AKT_LIVER/t-AKT_3h_liver.tif]

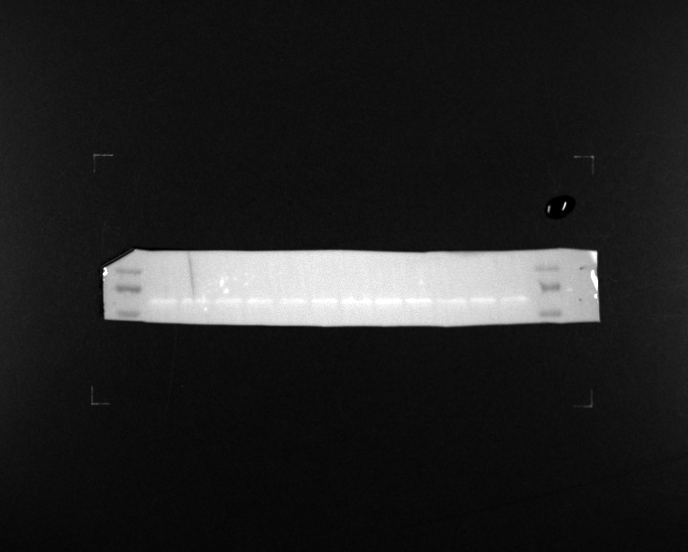

Supplement: Supplementary file 1 [file DataSheet1.zip › AKT_LIVER/t-AKT_3h_liver_bright field.tif]

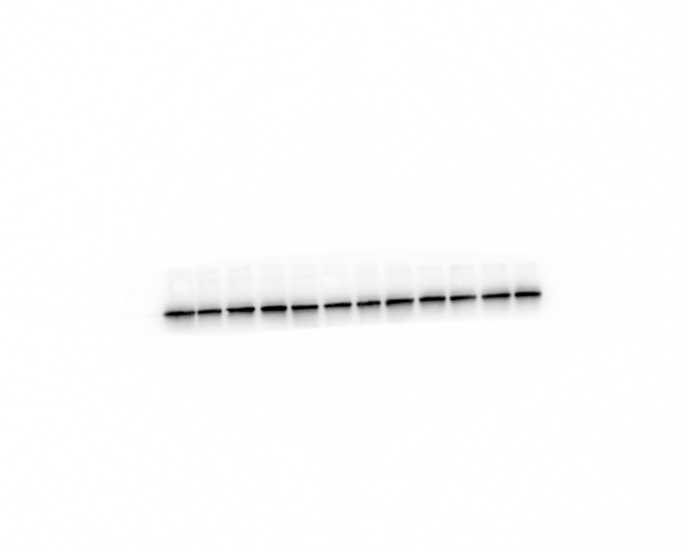

Supplement: Supplementary file 1 [file DataSheet1.zip › AKT_LIVER/t-AKT_6h_liver.tif]

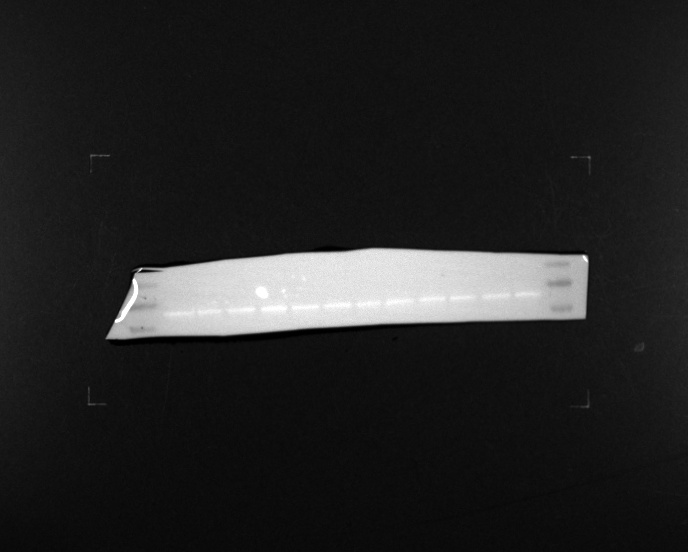

Supplement: Supplementary file 1 [file DataSheet1.zip › AKT_LIVER/t-AKT_6h_liver_bright field.tif]

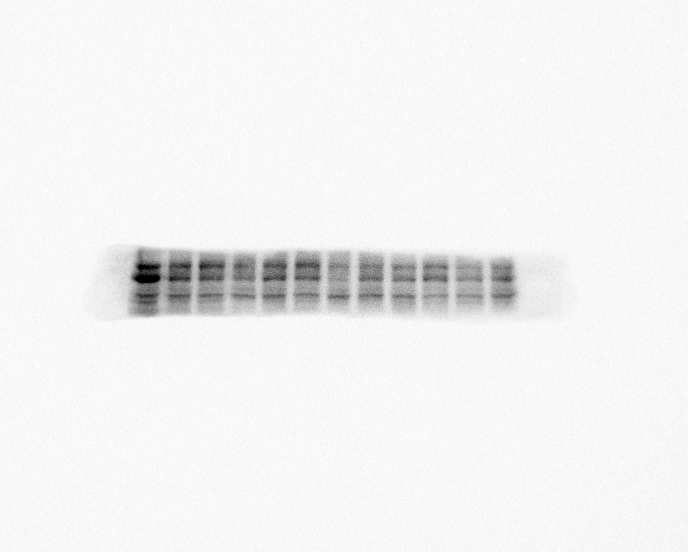

Supplement: Supplementary file 1 [file DataSheet1.zip › AMPK_Hypothalamus/p-AMPK_1h_hypothalamus.tif]

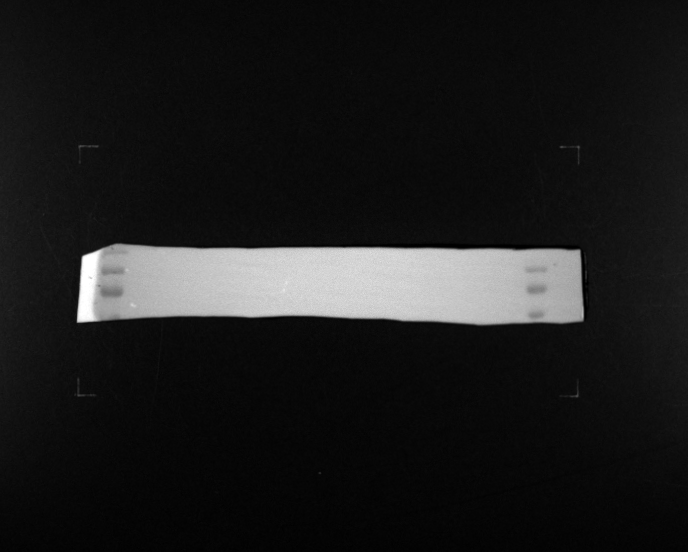

Supplement: Supplementary file 1 [file DataSheet1.zip › AMPK_Hypothalamus/p-AMPK_1h_hypothalamus_bright field.tif]

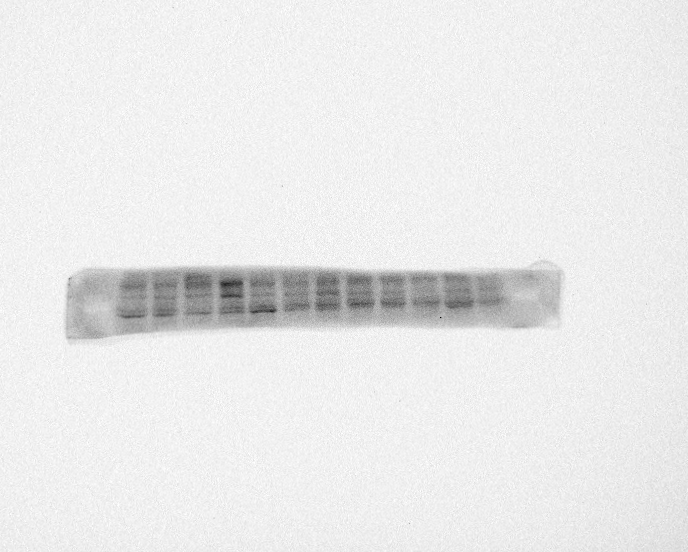

Supplement: Supplementary file 1 [file DataSheet1.zip › AMPK_Hypothalamus/p-AMPK_24h_hypothalamus.tif]

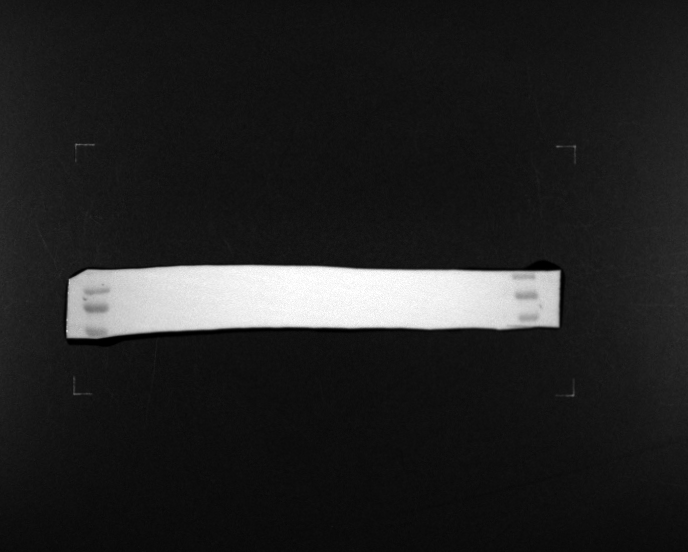

Supplement: Supplementary file 1 [file DataSheet1.zip › AMPK_Hypothalamus/p-AMPK_24h_hypothalamus_bright field.tif]

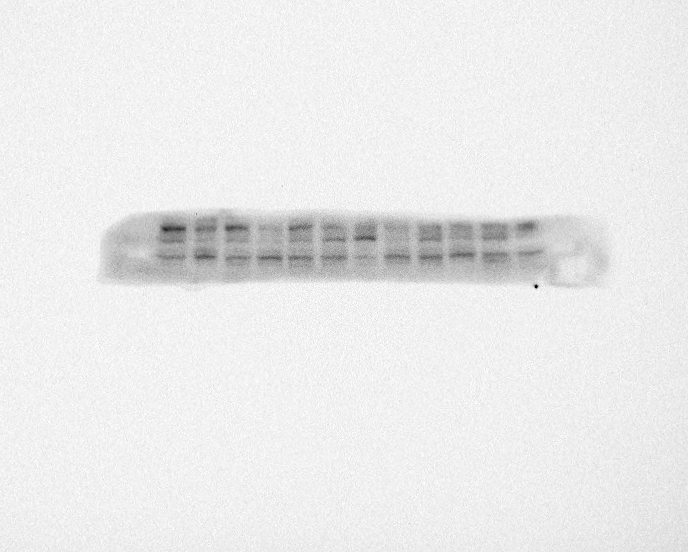

Supplement: Supplementary file 1 [file DataSheet1.zip › AMPK_Hypothalamus/p-AMPK_3h_hypothalamus.tif]

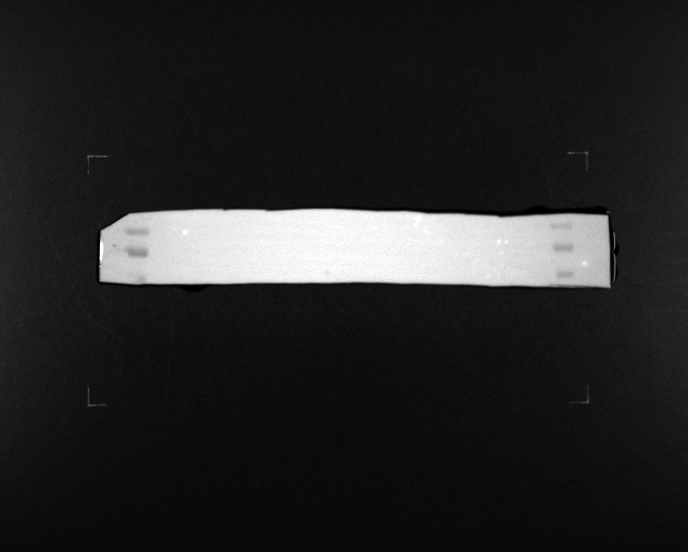

Supplement: Supplementary file 1 [file DataSheet1.zip › AMPK_Hypothalamus/p-AMPK_3h_hypothalamus_bright field.tif]

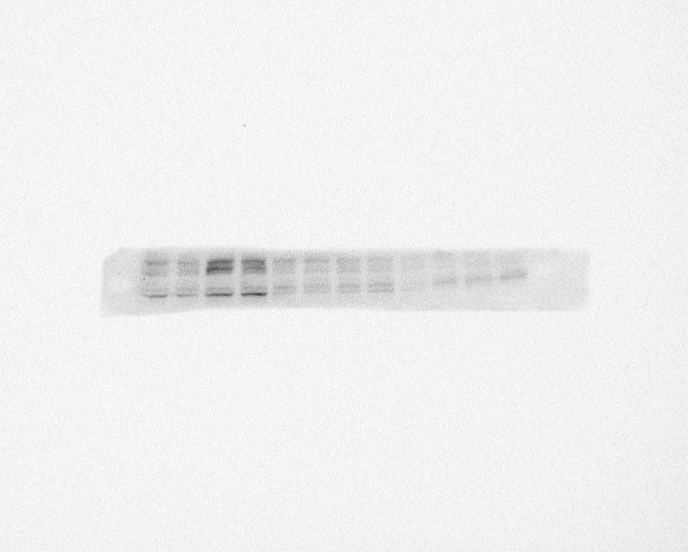

Supplement: Supplementary file 1 [file DataSheet1.zip › AMPK_Hypothalamus/p-AMPK_6h_hypothalamus.tif]

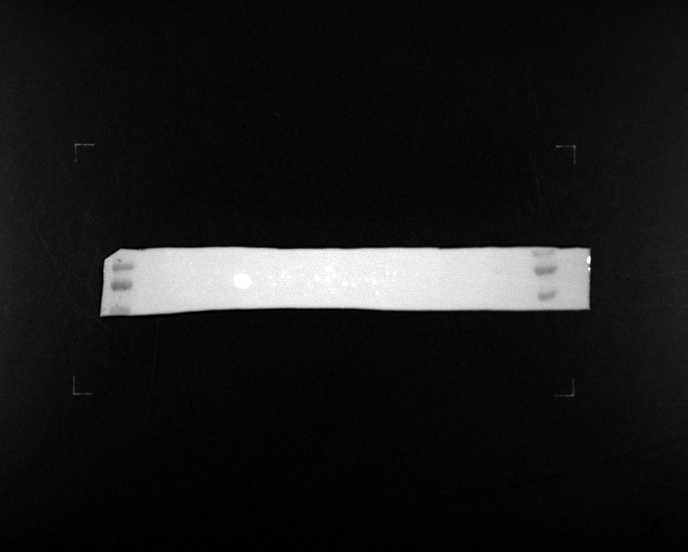

Supplement: Supplementary file 1 [file DataSheet1.zip › AMPK_Hypothalamus/p-AMPK_6h_hypothalamus_bright field.tif]

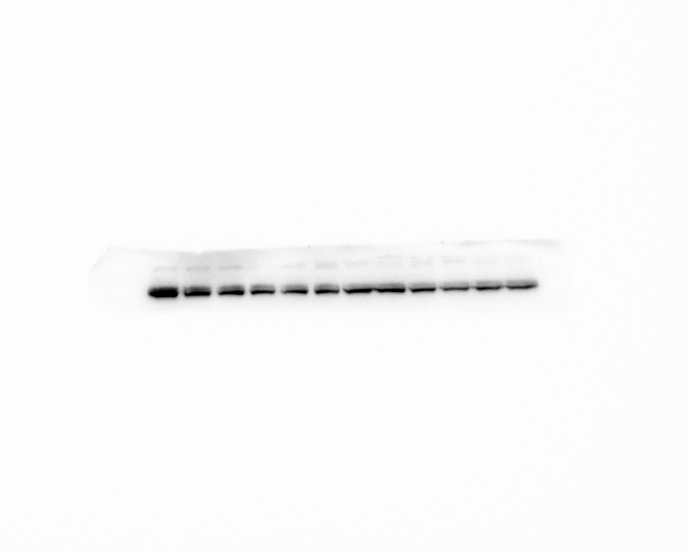

Supplement: Supplementary file 1 [file DataSheet1.zip › AMPK_Hypothalamus/t-AMPK_1h_hypothalamus.tif]

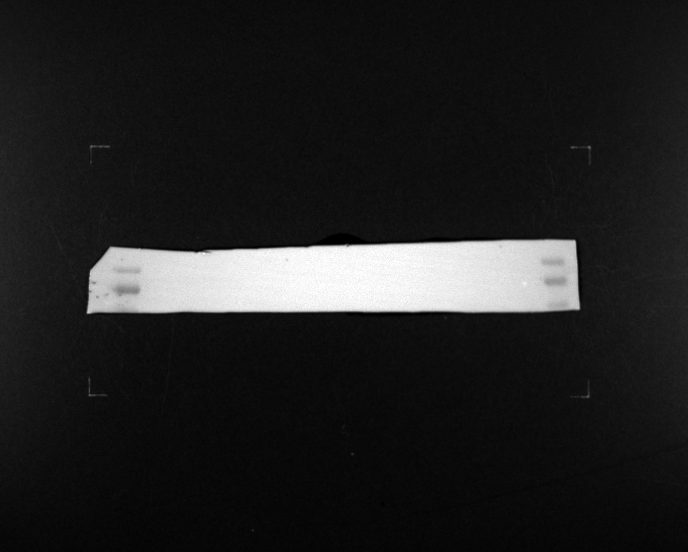

Supplement: Supplementary file 1 [file DataSheet1.zip › AMPK_Hypothalamus/t-AMPK_1h_hypothalamus_bright field.tif]

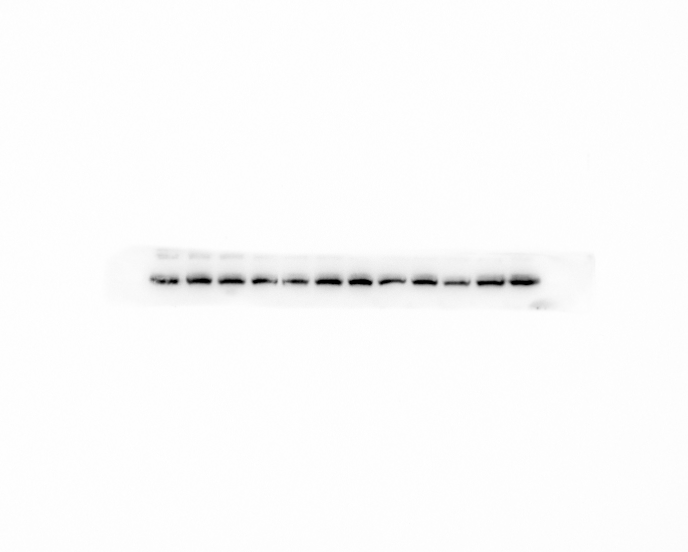

Supplement: Supplementary file 1 [file DataSheet1.zip › AMPK_Hypothalamus/t-AMPK_24h_hypothalamus.tif]

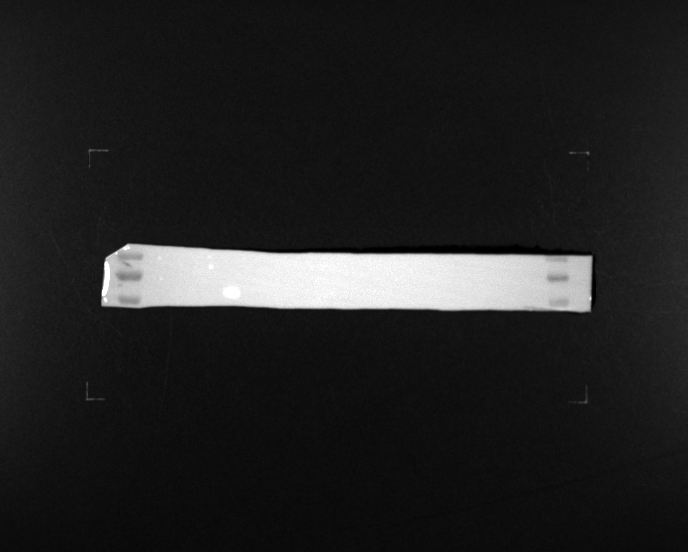

Supplement: Supplementary file 1 [file DataSheet1.zip › AMPK_Hypothalamus/t-AMPK_24h_hypothalamus_bright field.tif]

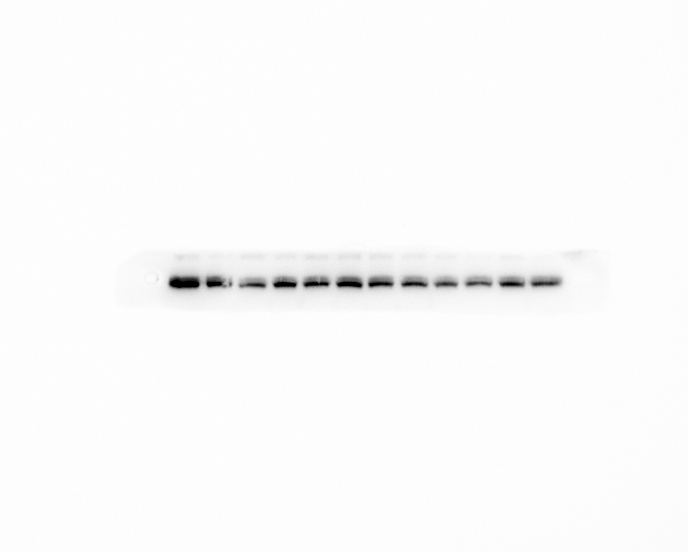

Supplement: Supplementary file 1 [file DataSheet1.zip › AMPK_Hypothalamus/t-AMPK_3h_hypothalamus.tif]

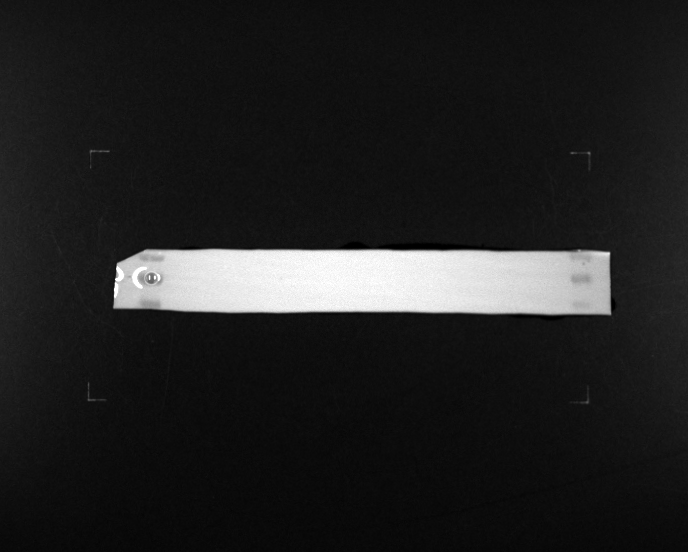

Supplement: Supplementary file 1 [file DataSheet1.zip › AMPK_Hypothalamus/t-AMPK_3h_hypothalamus_bright field.tif]

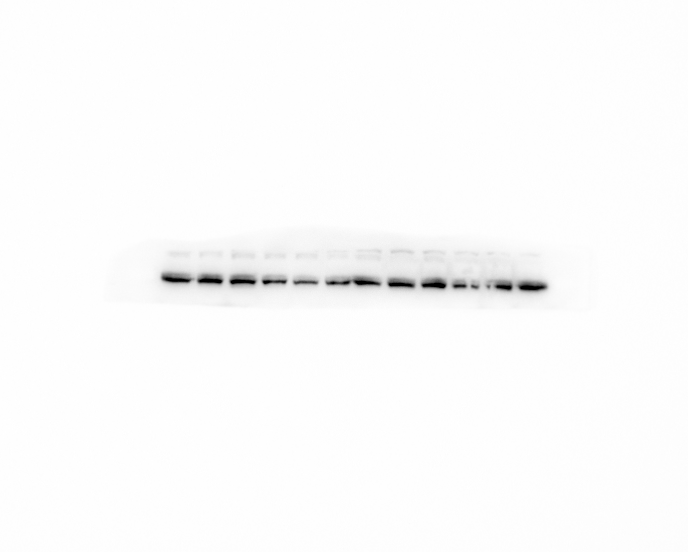

Supplement: Supplementary file 1 [file DataSheet1.zip › AMPK_Hypothalamus/t-AMPK_6h_hypothalamus.tif]

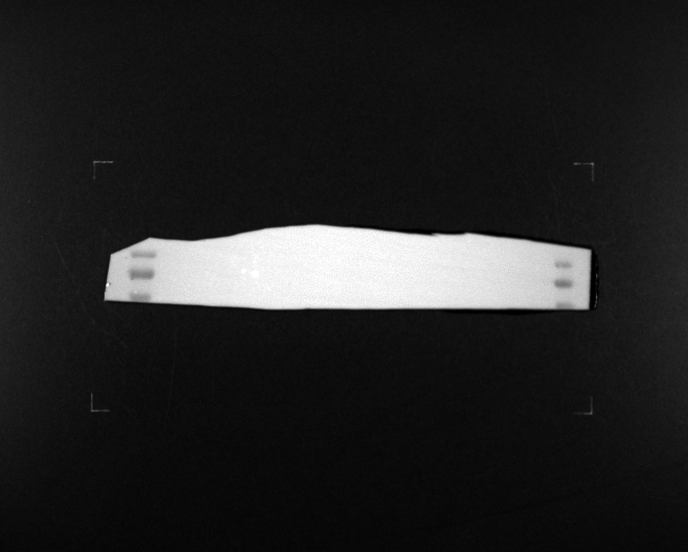

Supplement: Supplementary file 1 [file DataSheet1.zip › AMPK_Hypothalamus/t-AMPK_6h_hypothalamus_bright field.tif]

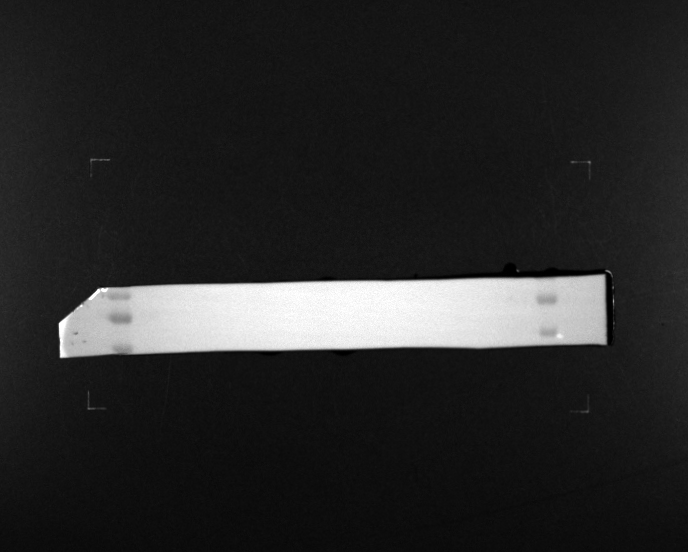

Supplement: Supplementary file 1 [file DataSheet1.zip › AMPK_LIVER/p-AMPK_1h_liver bright field.tif]

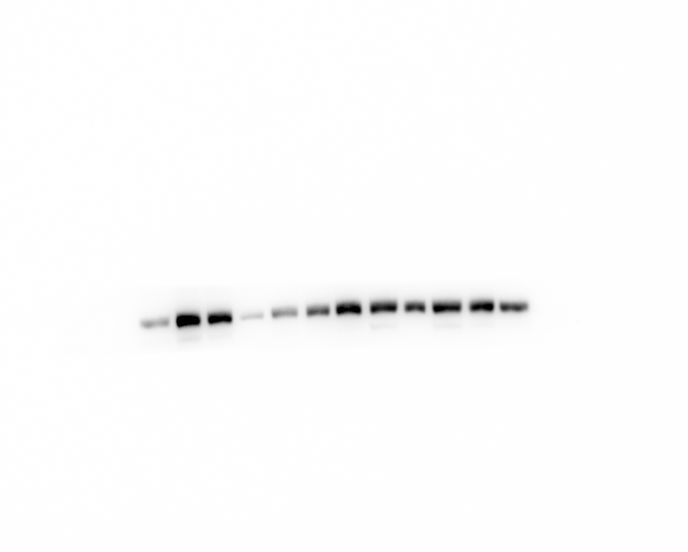

Supplement: Supplementary file 1 [file DataSheet1.zip › AMPK_LIVER/p-AMPK_1h_liver.tif]

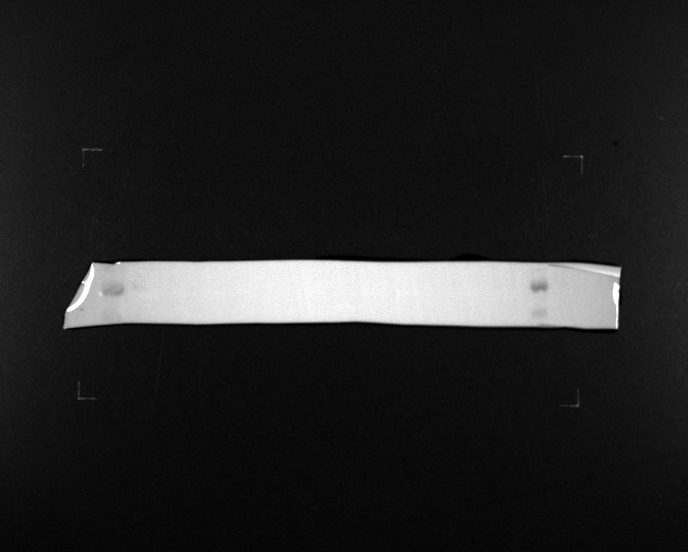

Supplement: Supplementary file 1 [file DataSheet1.zip › AMPK_LIVER/p-AMPK_24h_liver bright field.tif]

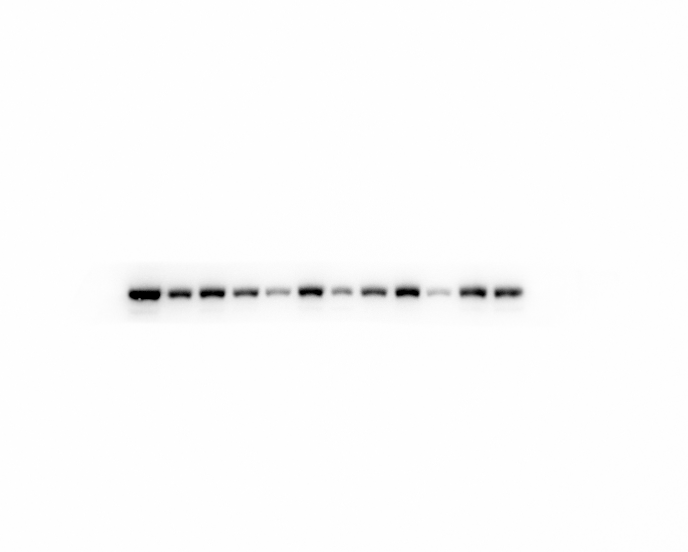

Supplement: Supplementary file 1 [file DataSheet1.zip › AMPK_LIVER/p-AMPK_24h_liver.tif]

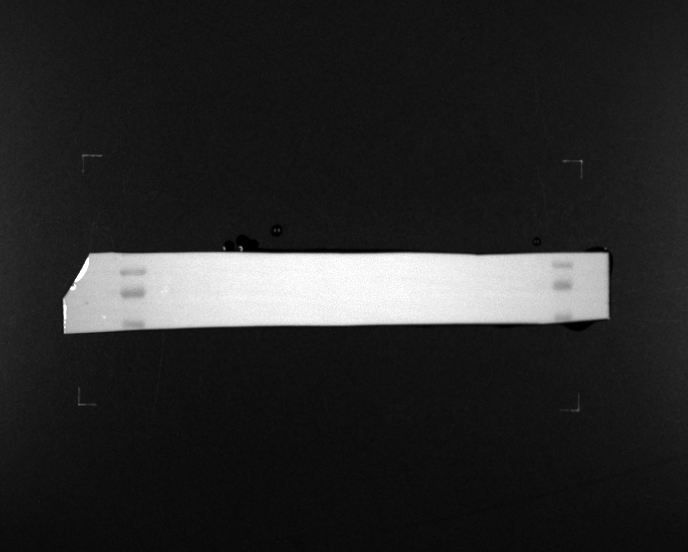

Supplement: Supplementary file 1 [file DataSheet1.zip › AMPK_LIVER/p-AMPK_3h_liver bright field.tif]

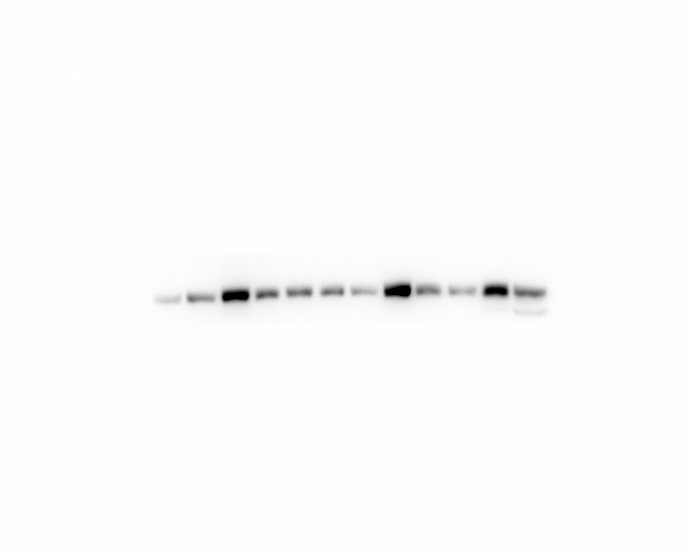

Supplement: Supplementary file 1 [file DataSheet1.zip › AMPK_LIVER/p-AMPK_3h_liver.tif]

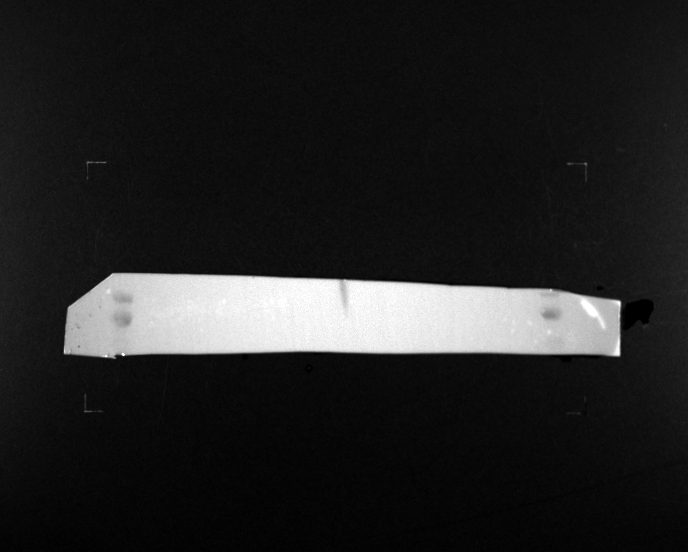

Supplement: Supplementary file 1 [file DataSheet1.zip › AMPK_LIVER/p-AMPK_6h_liver bright field.tif]

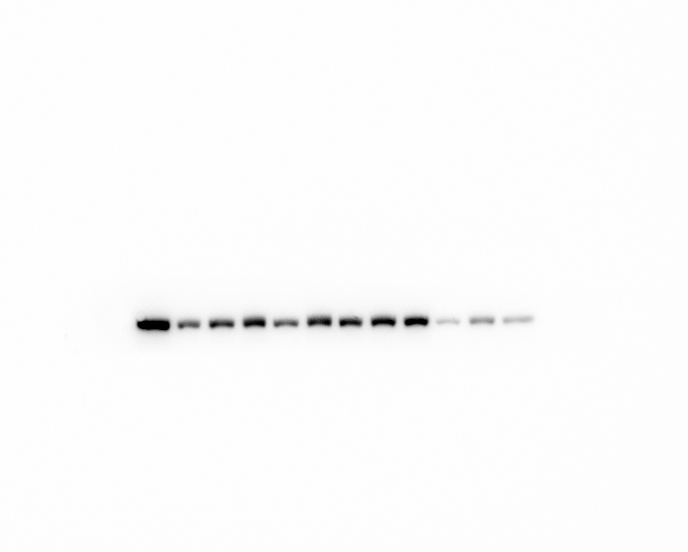

Supplement: Supplementary file 1 [file DataSheet1.zip › AMPK_LIVER/p-AMPK_6h_liver.tif]

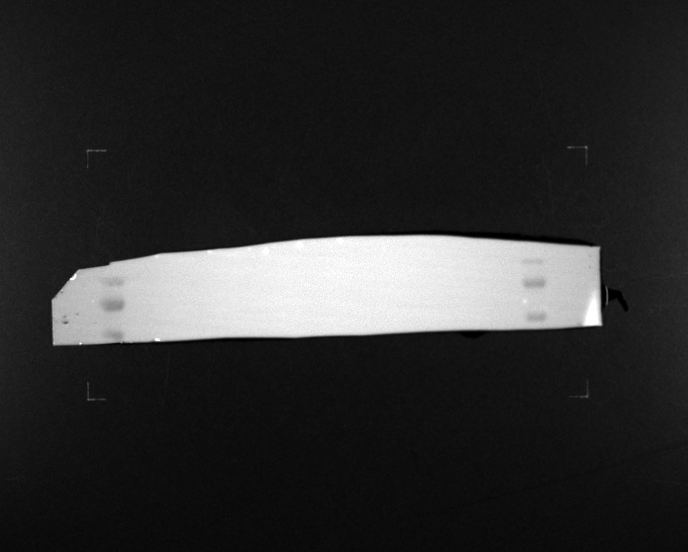

Supplement: Supplementary file 1 [file DataSheet1.zip › AMPK_LIVER/t-AMPK_1h_liver bright field.tif]

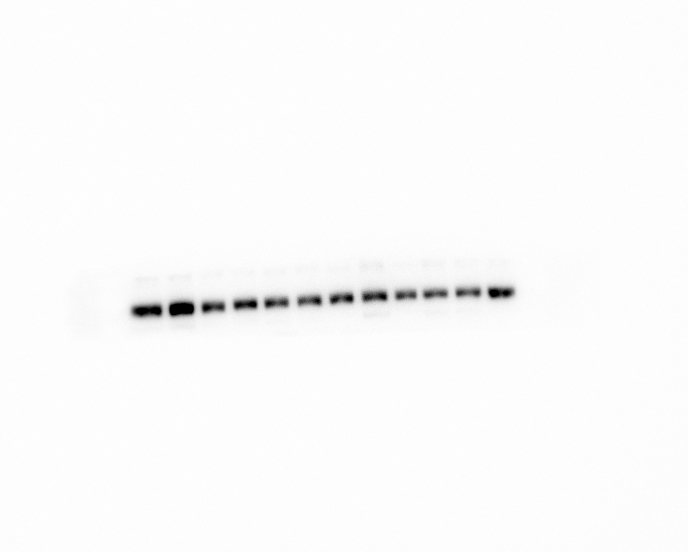

Supplement: Supplementary file 1 [file DataSheet1.zip › AMPK_LIVER/t-AMPK_1h_liver.tif]

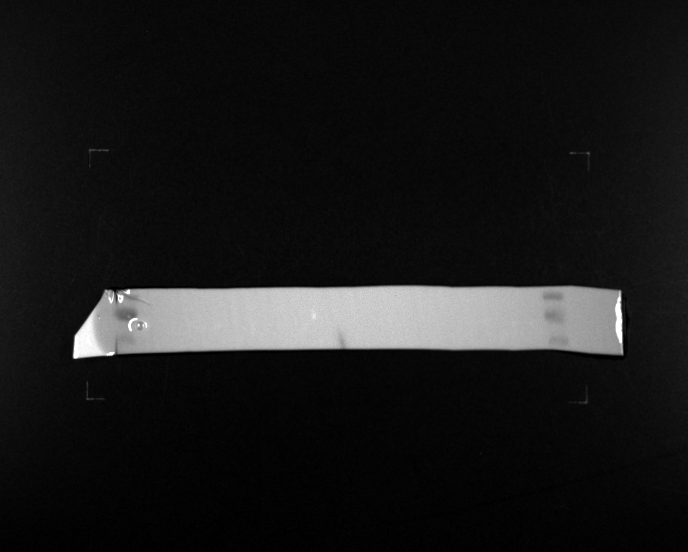

Supplement: Supplementary file 1 [file DataSheet1.zip › AMPK_LIVER/t-AMPK_24h_liver bright field.tif]

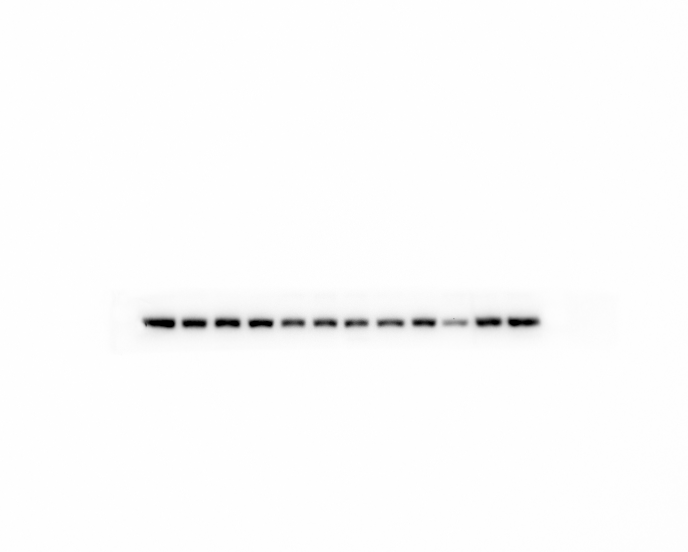

Supplement: Supplementary file 1 [file DataSheet1.zip › AMPK_LIVER/t-AMPK_24h_liver.tif]

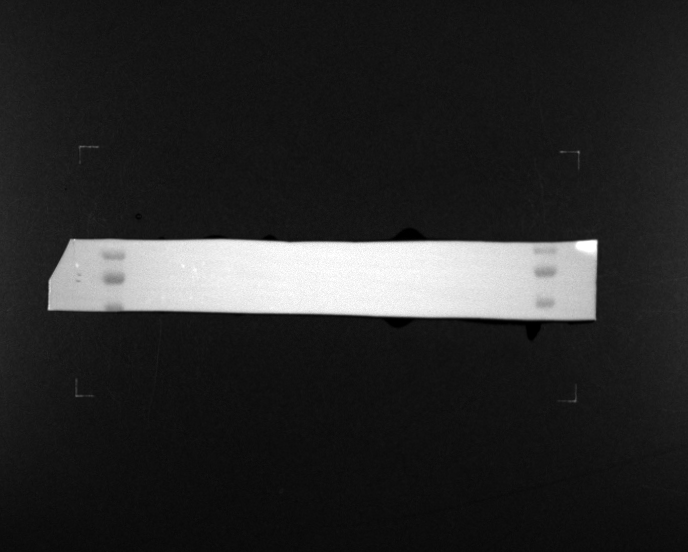

Supplement: Supplementary file 1 [file DataSheet1.zip › AMPK_LIVER/t-AMPK_3h_liver bright field.tif]

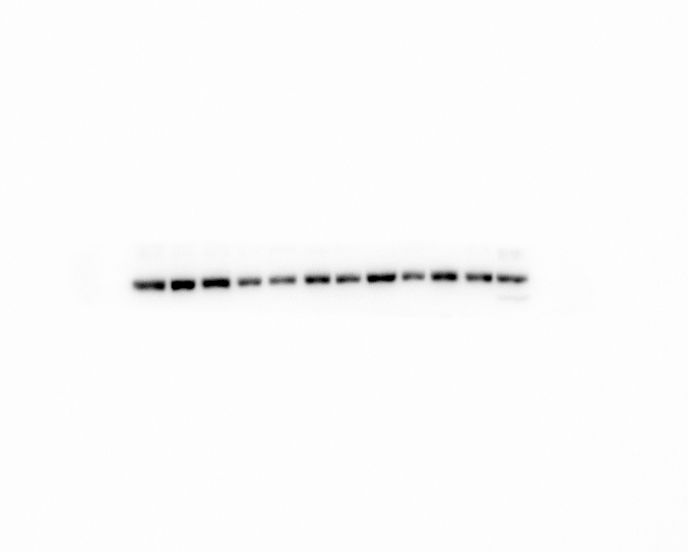

Supplement: Supplementary file 1 [file DataSheet1.zip › AMPK_LIVER/t-AMPK_3h_liver.tif]

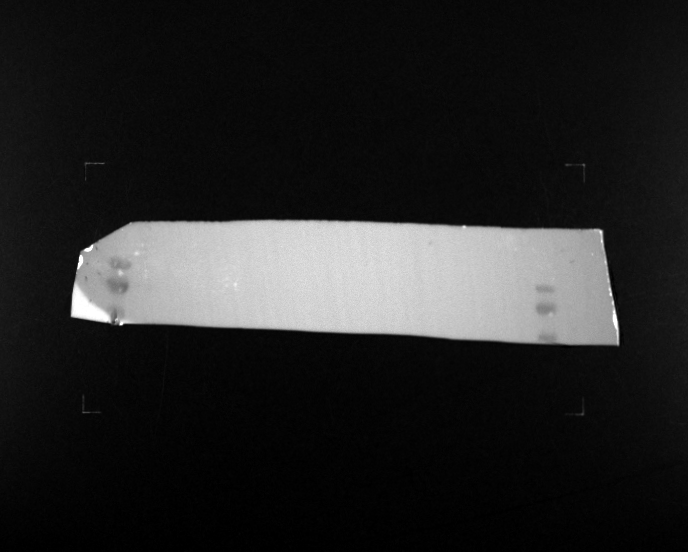

Supplement: Supplementary file 1 [file DataSheet1.zip › AMPK_LIVER/t-AMPK_6h_liver bright field.tif]

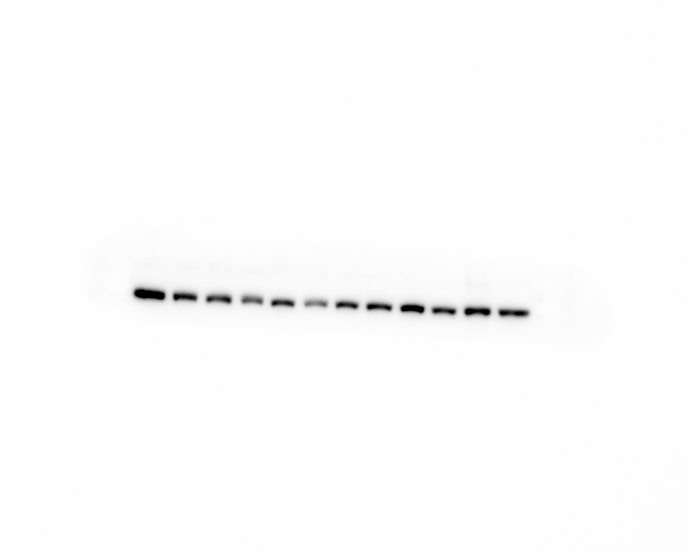

Supplement: Supplementary file 1 [file DataSheet1.zip › AMPK_LIVER/t-AMPK_6h_liver.tif]

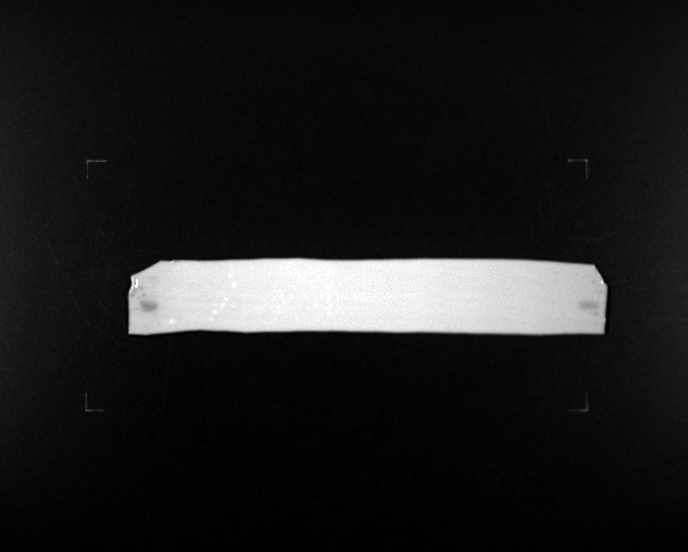

Supplement: Supplementary file 1 [file DataSheet1.zip › β-actin_Hypothalamus/β-actin_1h_hypothalamus bright field.tif]

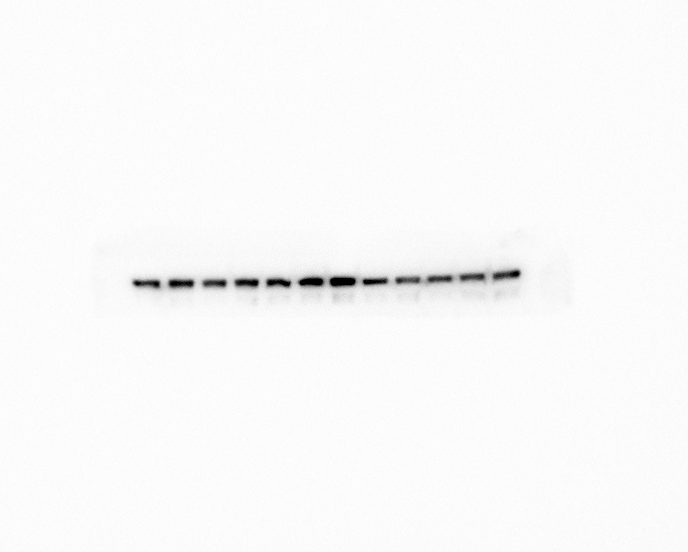

Supplement: Supplementary file 1 [file DataSheet1.zip › β-actin_Hypothalamus/β-actin_1h_hypothalamus.tif]

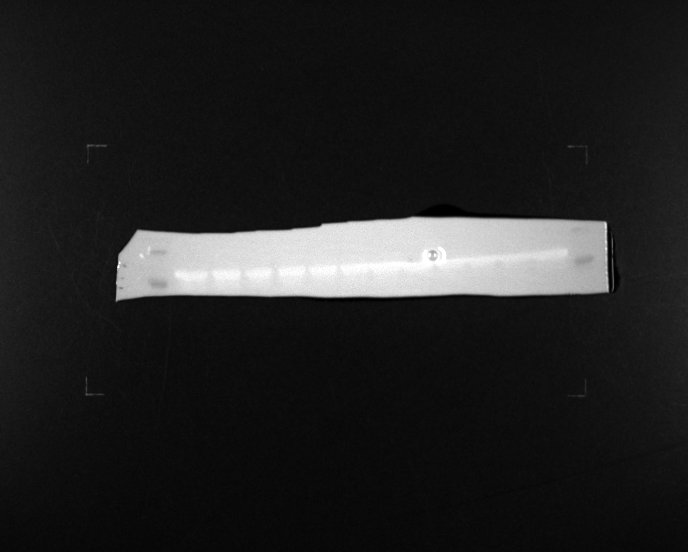

Supplement: Supplementary file 1 [file DataSheet1.zip › β-actin_Hypothalamus/β-actin_24h_hypothalamus bright field.tif]

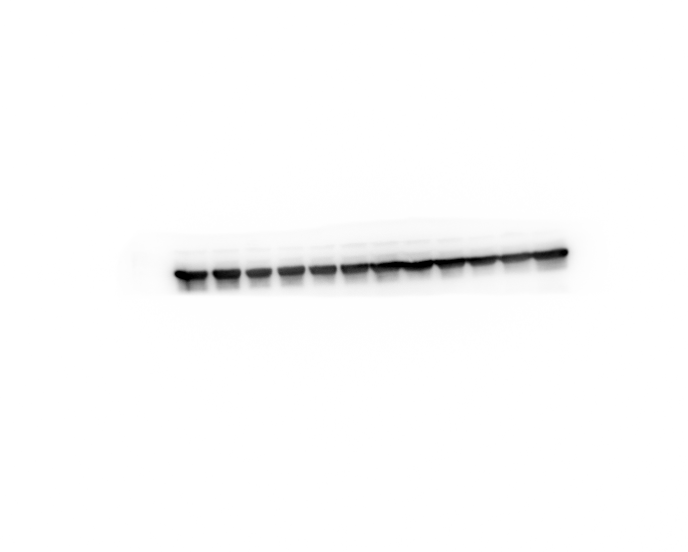

Supplement: Supplementary file 1 [file DataSheet1.zip › β-actin_Hypothalamus/β-actin_24h_hypothalamus.tif]

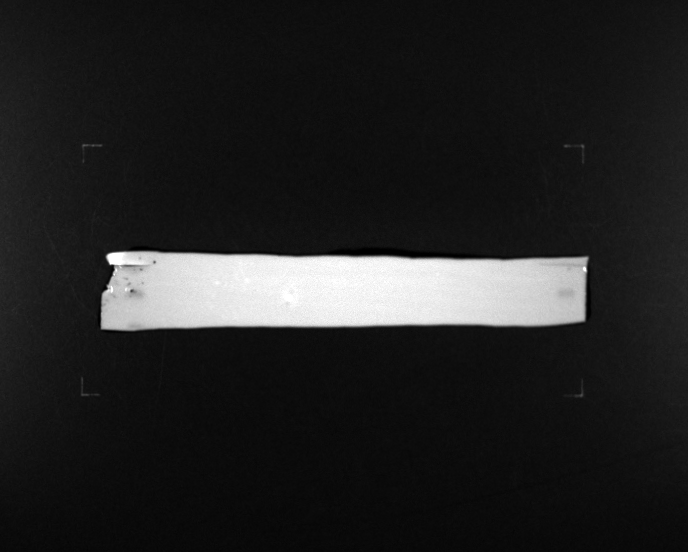

Supplement: Supplementary file 1 [file DataSheet1.zip › β-actin_Hypothalamus/β-actin_3h_hypothalamus bright field.tif]

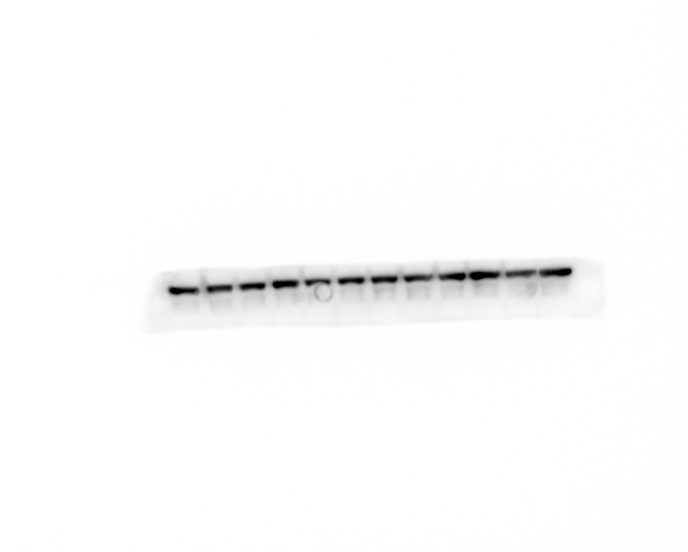

Supplement: Supplementary file 1 [file DataSheet1.zip › β-actin_Hypothalamus/β-actin_3h_hypothalamus.tif]

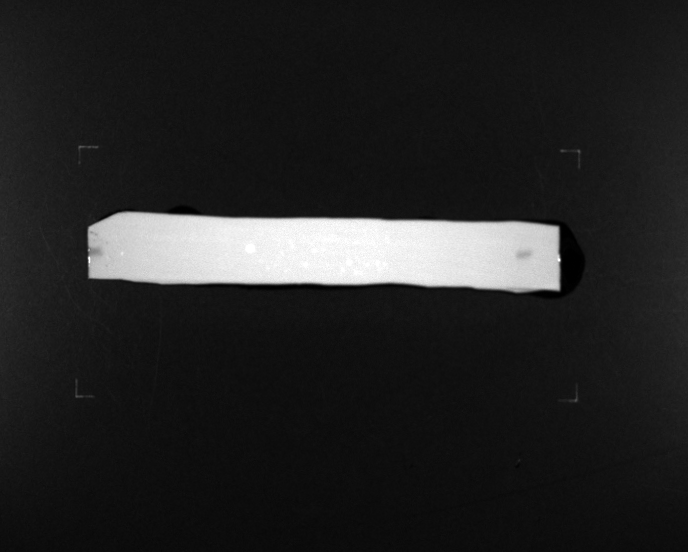

Supplement: Supplementary file 1 [file DataSheet1.zip › β-actin_Hypothalamus/β-actin_6h_hypothalamus bright field.tif]

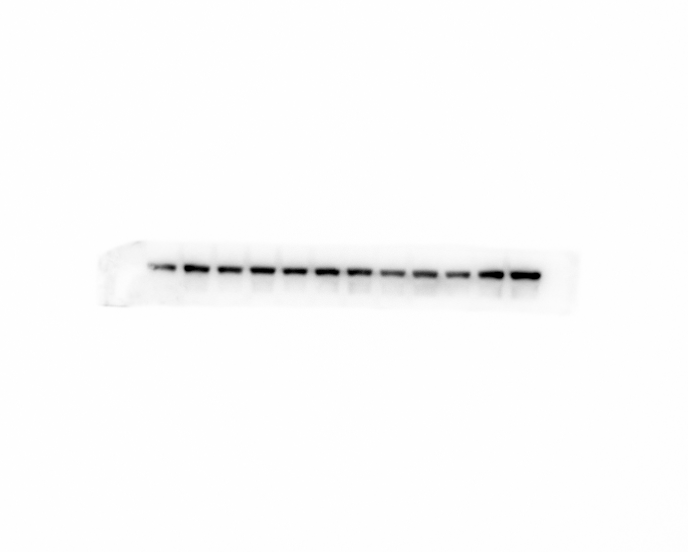

Supplement: Supplementary file 1 [file DataSheet1.zip › β-actin_Hypothalamus/β-actin_6h_hypothalamus.tif]

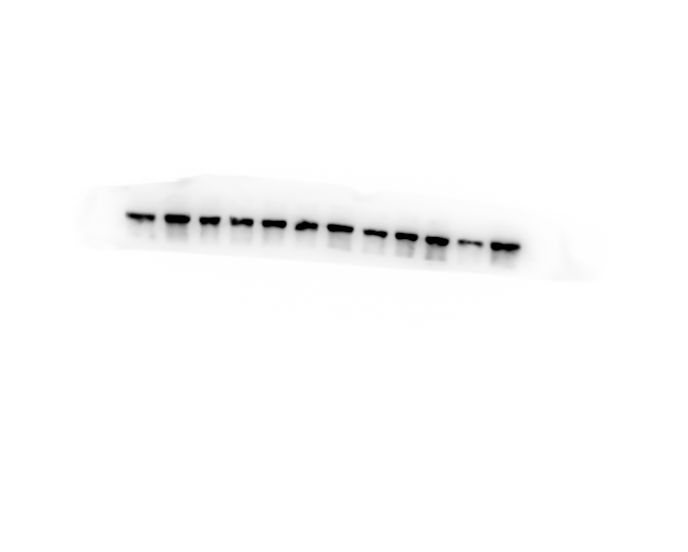

Supplement: Supplementary file 1 [file DataSheet1.zip › β-actin_LIVER/β-actin_1h_liver.tif]

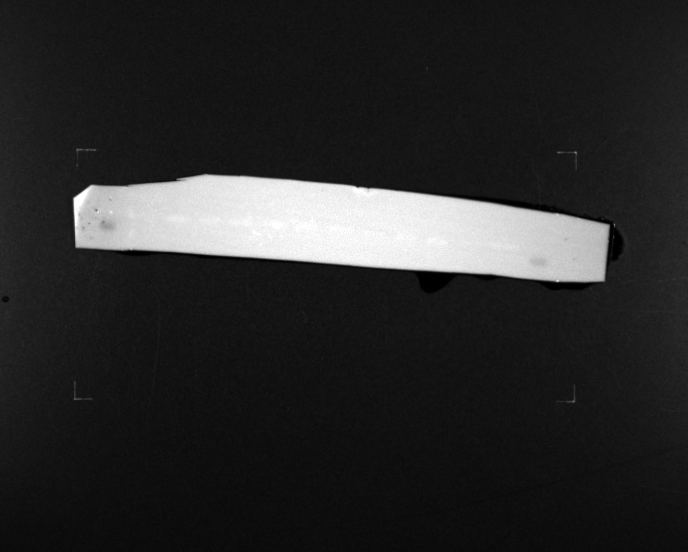

Supplement: Supplementary file 1 [file DataSheet1.zip › β-actin_LIVER/β-actin_1h_liver_bright field.tif]

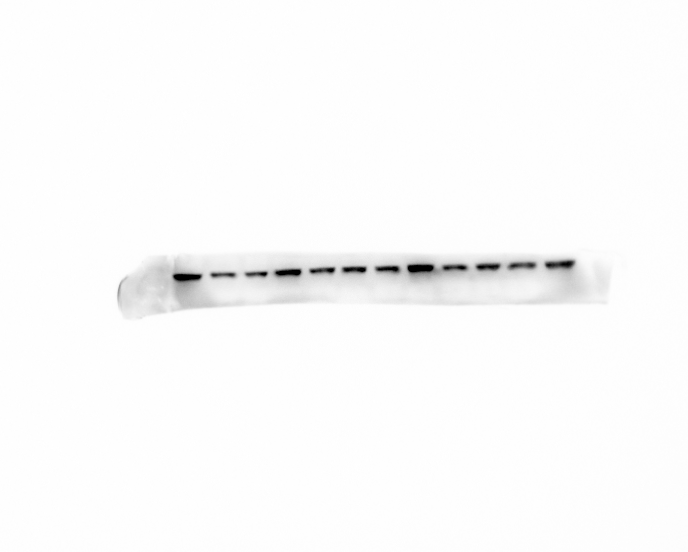

Supplement: Supplementary file 1 [file DataSheet1.zip › β-actin_LIVER/β-actin_24h_liver.tif]

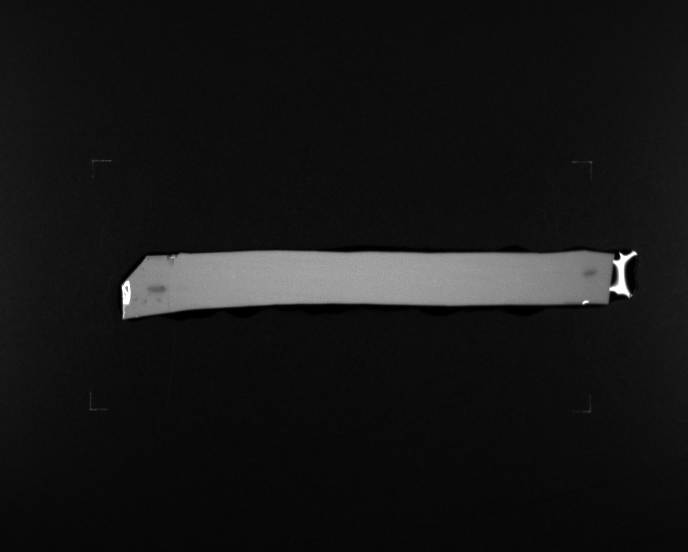

Supplement: Supplementary file 1 [file DataSheet1.zip › β-actin_LIVER/β-actin_24h_liver_bright field.tif]

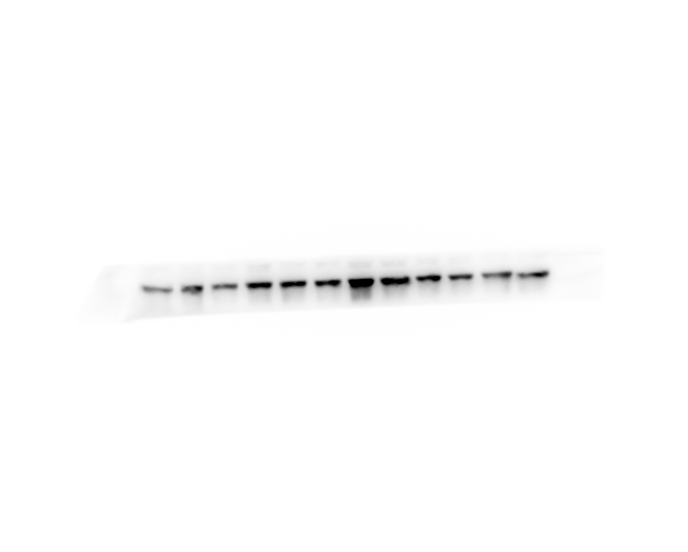

Supplement: Supplementary file 1 [file DataSheet1.zip › β-actin_LIVER/β-actin_3h_liver.tif]

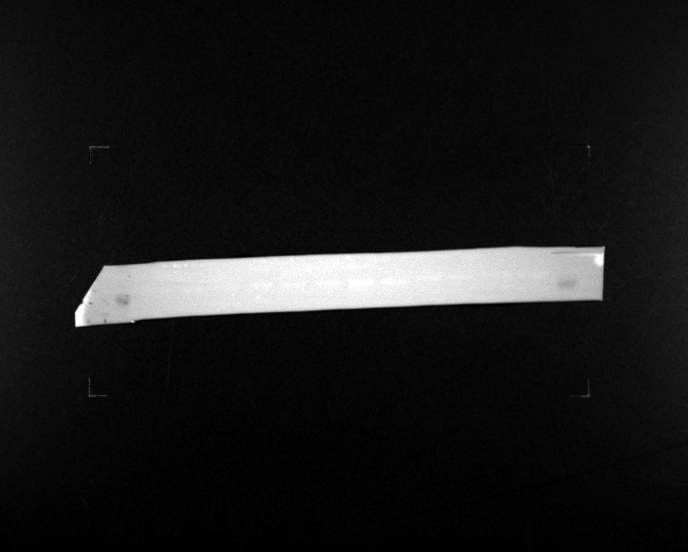

Supplement: Supplementary file 1 [file DataSheet1.zip › β-actin_LIVER/β-actin_3h_liver_bright field.tif]

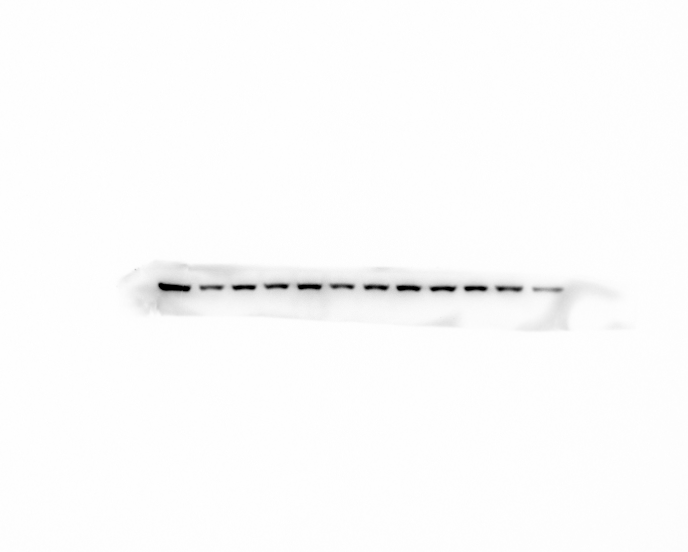

Supplement: Supplementary file 1 [file DataSheet1.zip › β-actin_LIVER/β-actin_6h_liver.tif]

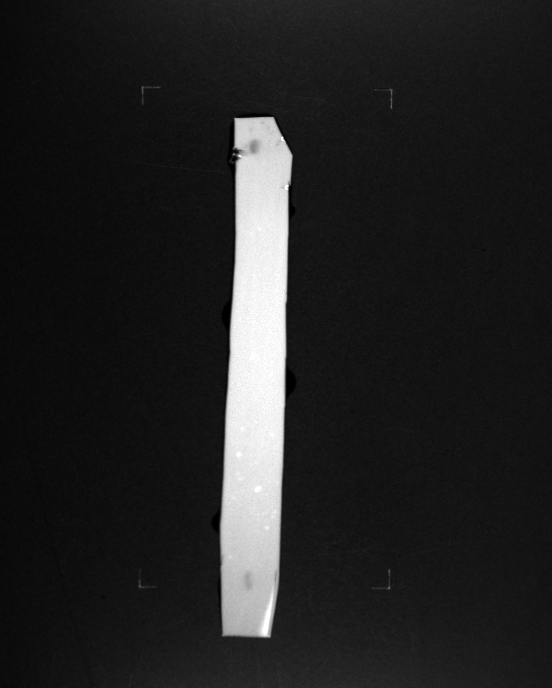

Supplement: Supplementary file 1 [file DataSheet1.zip › β-actin_LIVER/β-actin_6h_liver_bright field.tif]
